# Lattice oxygen activation enabled by high-valence metal sites for enhanced water oxidation

**DOI:** 10.1038/s41467-020-17934-7

**Published:** 2020-08-13

**Authors:** Ning Zhang, Xiaobin Feng, Dewei Rao, Xi Deng, Lejuan Cai, Bocheng Qiu, Ran Long, Yujie Xiong, Yang Lu, Yang Chai

**Affiliations:** 1 Department of Applied Physics, The Hong Kong Polytechnic University, Hung Hom, Kowloon, Hong Kong, P. R. China; 2 The Hong Kong Polytechnic University Shenzhen Research Institute, 518057 Shenzhen, P. R. China; 3 Department of Mechanical Engineering, City University of Hong Kong, Kowloon, Hong Kong, P. R. China; 4 Nano-Manufacturing Laboratory (NML), Shenzhen Research Institute of City University of Hong Kong, 518057 Shenzhen, P. R. China; 5 School of Materials Science and Engineering, Jiangsu University, 212013 Zhenjiang, Jiangsu P. R. China; 6 Hefei National Laboratory for Physical Sciences at the Microscale, iChEM (Collaborative Innovation Center of Chemistry for Energy Materials), School of Chemistry and Materials Science, National Synchrotron Radiation Laboratory, University of Science and Technology of China, 230026 Hefei, Anhui P. R. China

**Keywords:** Catalytic mechanisms, Density functional theory, Electrocatalysis

## Abstract

Anodic oxygen evolution reaction (OER) is recognized as kinetic bottleneck in water electrolysis. Transition metal sites with high valence states can accelerate the reaction kinetics to offer highly intrinsic activity, but suffer from thermodynamic formation barrier. Here, we show subtle engineering of highly oxidized Ni^4+^ species in surface reconstructed (oxy)hydroxides on multicomponent FeCoCrNi alloy film through interatomically electronic interplay. Our spectroscopic investigations with theoretical studies uncover that Fe component enables the formation of Ni^4+^ species, which is energetically favored by the multistep evolution of Ni^2+^→Ni^3+^→Ni^4+^. The dynamically constructed Ni^4+^ species drives holes into oxygen ligands to facilitate intramolecular oxygen coupling, triggering lattice oxygen activation to form Fe-Ni dual-sites as ultimate catalytic center with highly intrinsic activity. As a result, the surface reconstructed FeCoCrNi OER catalyst delivers outstanding mass activity and turnover frequency of 3601 A g_metal_^−1^ and 0.483 s^−1^ at an overpotential of 300 mV in alkaline electrolyte, respectively.

## Abstract (web summary)

Electrocatalytic water oxidation is facilitated by high valence states, but these are challenging to achieve at low applied potentials. Here, authors report a multicomponent FeCoCrNi alloy with dynamically formed Ni^4+^ species to offer high catalytic activity via lattice oxygen activation mechanism.

## Introduction

In recent few decades, there have been continuous developments towards water electrolysis, as the cathodically electrolytic hydrogen is proposed as an ideal energy carrier for the storage of sustainable but intermittent energy, such as wind and solar energy^1–3^. Current bottleneck mainly originates from four-electron process in anodic oxygen evolution reaction (OER), which requires large overpotential to surmount its sluggish reaction kinetics^4,5^. However, the high cost and instability of state-of-the-art iridium- and ruthenium-based electrocatalysts largely prevent their practical applications^6,7^. Earth-abundant catalysts based on 3*d* transition metals have been demonstrated as the promising alternatives for OER, especially in alkaline electrolyte^8–13^. Meanwhile, experimental and theoretical studies reach a consensus that late transition metals with high valence states exhibit superior activities^2,14–16^. The increased holes in *d*-band of highly oxidized metal species can enhance the covalency of metal–oxygen (M–O) bonds to promote the charge transfer^9,14^. More importantly, high valency typically induces the downshift of metal *d*-band to penetrate *p*-band of oxygen ligands^17^. The redox electrochemistry of oxygen ligands will be triggered by driving holes into the related oxygen *p*-band, making lattice oxygen atoms electrophilic to participate in water oxidation, so called lattice oxygen activation mechanism (LOM)^12,17,18^. This alternative pathway facilitates the direct lattice oxygen coupling (LOC) by sharing the ligand holes, thereby lowering the limiting energy barrier. Thus, we can expect that rationalization of LOM pathway with highly oxidized metal species provides a promising avenue to maximize efficiency of OER electrocatalysts. However, according to the Pourbaix diagrams, it usually requires more elevated potential to realize deep oxidation of metal species^15,16^, causing the thermodynamically unfavorable formation of highly oxidized metal species. Those disadvantages make LOM pathway unpredictable and hinder the exploitation of efficient OER electrocatalysts. Therefore, it is highly desirable to steer the highly oxidized metal species with minimizing their formation energy.

In general, the OER electrocatalysts undergo the surface reconstruction into (oxy)hydroxides, independent of initial composition and structure^19–21^, wherein the structural flexibility of (oxy)hydroxides enables the dynamic self-optimization of catalytically active sites. This dynamic reconstruction normally involves oxidation of metal sites, along with adsorption of oxygen species and/or deprotonation of hydroxyl. Therefore, the redox electrochemistry is directly related to the chemical affinity between metal sites and oxygen species, which can be subtly manipulated by the variation of electronic states^11,21–23^. Multimetal-based electrocatalysts typically endows more superior activities than single-metal catalysts, as the interatomically electronic interplay can efficiently modulate the electronic structure of metal sites that can hardly achieve for single-metal catalysts, offering an effective approach to tune the redox electrochemistry and engineer highly oxidized metal species^24–26^. Owing to their structural complicacy, it is still a challenging task to rationally design efficient multimetal OER catalysts with high valence metal sites and fundamentally understand their structure-activity correlation.

Multicomponent alloy (MCA) materials have recently received extensive attention because of their unique intrinsic properties^27,28^. The adjustable components make them feasible to serve as templates for multimetal-based electrocatalysts. By rational engineering, it enables the formation of highly oxidized metal species and tune on LOM. Here, we demonstrate that an FeCoCrNi MCA film can dynamically form highly oxidized metal species during OER process with decreasing formation energy after undergoing irreversible surface reconstruction. Our spectroscopic investigations and theoretical simulations reveal that the interatomically electronic interplay in surface reconstructed multimetal (oxy)hydroxide (MO_*x*_H_*y*_) plays a key role on subtly engineering highly oxidized Ni^4+^ species to favor LOM pathway. Fe component induces electron depletion in Ni species to ensure the dynamic formation of Ni^4+^ species. Meanwhile, a multistep evolution of Ni^2+^→Ni^3+^→Ni^4+^ lowers the overall energy barrier of Ni^4+^ formation, facilitated by pre-adsorbed bridging hydroxyl at Ni-Co site. The constructed Ni^4+^ species drives holes into oxygen ligands to tune on intramolecular LOC via Mars-van Krevelen-like mechanism, thereby evoking LOM pathway. A unique Fe–Ni dual-site with oxygen vacancies (OVs) is formed after desorbing the coupled peroxo-like oxygen species to serve as ultimate catalytic center, substantially promoting OER activity.

## Results

### Preparation and characterizations of catalytic systems

**Fig. 1:**
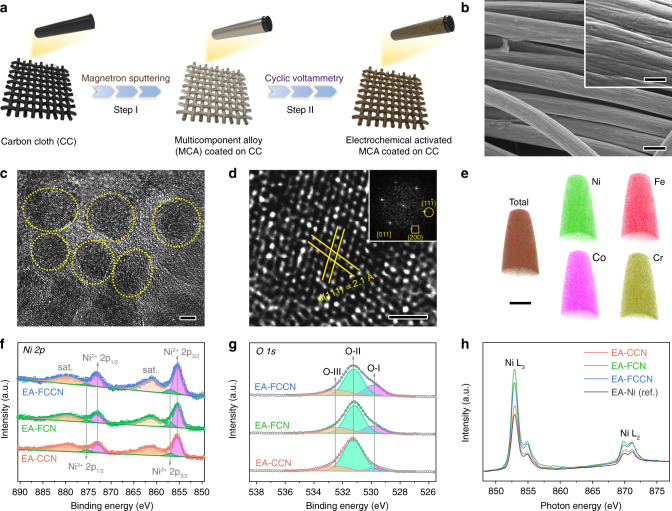
Characterizations of as-prepared electrocatalysts. **a** Schematic illustration of the electrocatalyst preparation. **b**–**e** Structural characterizations of pristine FeCoCrNi MCA film. **b** SEM image. Scan bar, 10 μm. Insert shows the Partially magnified SEM image. Scan bar, 1 μm. **c** Magnified TEM image. Scan bar, 2 nm. **d** HRTEM image. Scan bar, 1 nm. Insert is the corresponding FFT pattern. **e** 3D APT probing reconstruction images of Ni (green), Fe (red), Co (pink), and Cr (olive) atom positions. Scan bar, 20 nm. **f**–**h** Electronic characterizations of electrochemical activated MCA films. High-resolution (**f**) Ni 2*p* and (**g**) O 1*s* XPS spectra. **h** Ni *L*-edge sXAS spectra.

A two-step procedure was used to prepare the multimetal-based electrocatalysts, as shown in Fig. 1a. We firstly employed magnetron sputtering method (Supplementary Fig. 1)^29,30^ to deposit MCA films (quaternary FeCoCrNi and ternary FeCrNi and CoCrNi) on pretreated carbon clothes (CCs). Scanning electron microscopy (SEM) images (Fig. 1b and Supplementary Figs. 2 and 3) demonstrate that the films are tightly and uniformly coated on the surface of CCs. The characteristic peak at 2θ = 44.2° in X-ray diffraction (XRD) patterns (Supplementary Fig. 4) of MCA films can be indexed to (111) lattice planes of face-centered cubic (FCC) phase^30^. Transmission electron microscope (TEM) image of FeCoCrNi MCA sample (Supplementary Fig. 5) displays its polycrystalline feature, as the film is composed by interconnected nanocrystals with the size of ~5 nm (see magnified TEM image in Fig. 1c). The related High-resolution TEM (HRTEM) image together with corresponding fast Fourier transform (FFT) pattern (Fig. 1d) exhibit the ordered lattice fringes with a spacing of 2.1 Å, which can be assigned to the (111) planes, in good agreement with XRD peaks. To examine the elemental composition and distribution in MCA films, energy dispersive X-ray spectroscopy (EDS) mapping images were collected, which clearly demonstrate the uniformly elemental distribution throughout the measured region (Supplementary Fig. 6). Quantitative analysis reveals the elemental distribution is nominally equiatomic with slightly lower Cr concentration (*ca*. 80%), coinciding with inductively coupled plasma mass spectrometry (ICP-MS) results (Supplementary Fig. 7 and Supplementary Table 1). The homogenous elemental distribution is reaffirmed by 3D atomic-probe tomography (APT) in atomic scale^30^, where 3D APT probing reconstruction images (Fig. 1e) show negligible segregation and clustering. Besides, X-ray photoelectron spectroscopy (XPS) was adopted to characterize the electronic states of MCA films (Supplementary Figs. 8 and 9 and Supplementary Table 2). The MCA films exhibit the metallic feature with inevitable surface oxidation. The oxidized degree of various metal components is well distinguished and positively correlates to the related metal reduction potentials (Supplementary Table 3). More details are discussed in Supplementary Note 1.

We further activated MCA films using an electrochemical cyclic voltammetry (CV) scanning procedure (Supplementary Fig. 10a–c). The electrochemical activated FeCoCrNi, FeCrNi, and CoCrNi MCA films (namely, EA-FCCN, EA-FCN, and EA-CCN, respectively) were investigated comprehensively to resolve the morphologic and electronic structure information. SEM (Supplementary Fig. 10d–f) and TEM (Supplementary Fig. 11) images show rough surface along with numerous nanosheets, clarifying the irreversible reconstruction. Besides, XRD patterns (Supplementary Fig. 12) confirm the reconstruction into MO_*x*_H_*y*_, which is reasonable in an alkaline electrolyte^19–21^. The electrochemical activated MCA films were also studied by XPS to affirm the electronic states of metal components (Fig. 1f and Supplementary Fig. 13). The recorded spectrums are largely differentiated with those of pristine MCA films, where all of the metal components exhibit the thorough oxidation (deconvoluted results are summarized in Supplementary Table 4). Specifically, Ni 2*p* XPS spectra (Fig. 1f) indicates two dominant spin-orbit peaks of Ni^2+^ species in (oxy)hydroxide with secondary Ni^3+^ species. Differently, Co^3+^ species is dominant compared to Co^2+^ species, while Fe and Cr components are fully oxidized to M^3+^ species (see Supplementary Fig. 13 and Supplementary Note 2 for detailed discussions). Although we can still collect the signal of Cr species, the dramatically decreased intensity (less than 10% retaining, see Supplementary Fig. 15) indicates its leaching during electrochemical activation^31^. The removal of Cr component is probably ascribed to its early oxidation and amphoteric characteristics. Furthermore, we interpret that the reconstruction is a surface engineering without altering the bulk matrix, corroborated by depth-dependent XPS spectra with Ar^+^ plasma etching (Supplementary Figs. 14–16) and ICP-MS analysis (Supplementary Fig. 17). This surface reconstruction can retain the bulk conductivity of the alloy film, guaranteeing the effective bulk charge transfer during electrocatalytic process. Given the surface oxidation, we also collected O 1*s* XPS spectra (Fig. 1g). The deconvolution shows three characteristic peaks at 529.8, 531.3, and 532.6 eV (marked as O–I, O–II, and O–III), assignable to oxygen species in M–O, M–OH, and adsorbed H_2_O at OVs, respectively^13,32^. The dominant hydroxyl species (O–II peak) reaffirms the reconstructed MO_*x*_H_*y*_ on MCA films. It is noteworthy that both O–I and O–III peaks increase in Fe-alloyed films (Supplementary Fig. 18), unveiling that Fe component favors the dissociation of O–H bond and formation of OVs, which potentially benefits the catalytic activity.

Considering the surface reconstruction of MCA films, we further used a more surface sensitive technique—soft X-ray adsorption spectroscopy (sXAS) with total electron yield (TEY) mode (detecting depth is a few nanometers)—to probe the 3*d* electronic information of surface metal species^16,24,33^. Metal *L*-edge sXAS spectra derived from dipole-allowed *p* → *d* electron transition can sensitively monitor the unoccupied states in *d*-orbitals of transition metals, where lower electron density embodies inversely higher white line intensity in the spectra^10,25^. For our samples, Ni component demonstrates characteristic white line of Ni^2+^ species (Fig. 1h), while Co^3+^ and Fe^3+^ species are recognized to dominantly exist (Supplementary Fig. 19)^10,16,24,25^, matching the results of XPS spectra. More importantly, the intensities of white lines are distinctly varied, which implies the electronic modulation of surface metal species. Typically, *O*_h_-symmetric Metal atoms interact with bridging oxygen (*μ*–O) through *π*-donation in (oxy)hydroxide structures, forming M–O-M moiety (Supplementary Fig. 20a)^22^. Due to different electronic occupancy in *π*-symmetric *d*-orbitals (i.e., *t*_2g_-orbitals), partial electron transfer (PET) can be triggered through *μ*-O ligand, leading to the electron density fluctuation (Supplementary Fig. 20b)^22,25,34^. The strength of PET can be determined by the occupancy in *t*_2g_-orbitals, namely, Fe^3+^-O-Ni^2+^ > Fe^3+^-O-Co^3+^ > Co^3+^-O-Ni^2+^ (left metal atom serves as the electron acceptor, see details in Supplementary Note 3), which well coincides with varied white line intensities of sXAS spectra. Taking Ni species as an instance (Fig. 1h), the higher white line intensities of our samples related to pure Ni sample (EA-Ni) indicates the electron density decrease due to PET from Ni^2+^ to Fe^3+^/Co^3^. Furthermore, the stronger PET in Fe^3+^-O-Ni^2+^ moiety gives rise to more electron depletion in Ni^2+^ species, leading to the further increased white line intensities for EA-FCN and EA-FCCN related to EA-CCN. The interatomically electronic interplay through PET in M–O-M moiety can delocalize and redistribute the electrons in *d*-orbitals of surface metal atoms, thereby largely influencing the catalytic activity^25,34^.

### Evaluation of electrocatalytic OER activity

**Fig. 2:**
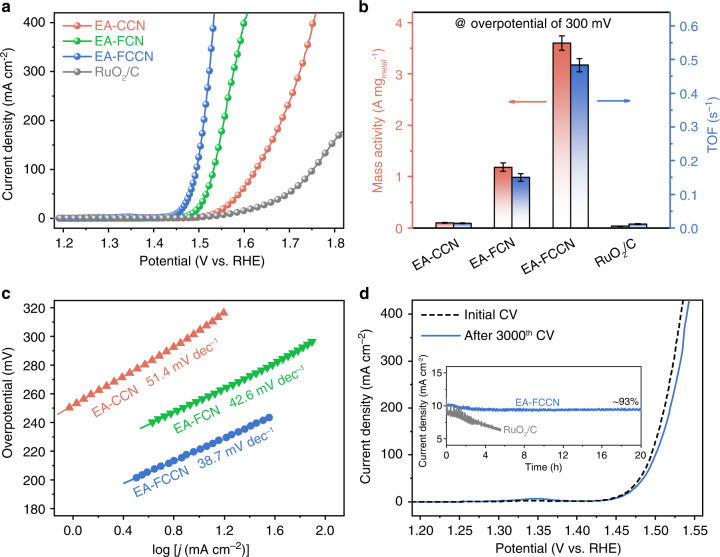
Electrochemical OER activity evaluations. **a** LSV polarization curves of various catalysts in O_2_-saturated 1-M KOH solution at a scan rate of 5 mV s^−1^. **b** Mass activities and TOFs of various catalysts at overpotential of 300 mV. **c** Tafel plots of various catalysts derived from polarization curves in Fig. 3a. **d** LSV polarization curves of EA-FCCN before and after 3000 CV cycles for stability test. Inset is 20-h chronoamperometric curves with the initial current density of 10 mA cm^−2^.

Figure 2a displays the collected linear sweep voltammogram (LSV) polarization curves of various catalysts, clearly showing that our electrochemical activated MCA films significantly outperform the benchmark RuO_2_ catalyst. An overpotential of 304 mV is required for EA-CCN to achieve a current density of 10 mA cm^−2^ (normalized by geometrical area of electrode), which further decreases to 255 and 221 mV for EA-FCN and EA-FCCN, respectively (Supplementary Fig. 21). The substantially improved activity implies that the interatomically electronic interplay in surface MO_*x*_H_*y*_ plays a pivotal role on water oxidation^24–26^. It is noteworthy that EA-FCCN only requires the overpotentials of 281 and 301 mV to reach the large current densities of 200 and 400 mA cm^−2^, respectively, exhibiting its superiority as the potential OER electrocatalyst candidate for practical applications. To assess the activity of our catalysts more fairly, specific activities were normalized by the electrochemically active surface areas (ECSAs), which were estimated by double-layer capacitances (*C*_dl_s) (see Supplementary Fig. 22 and Supplementary Table 5). As displayed in Supplementary Fig. 23, although ECSA of EA-FCCN is relative larger, the determined specific activity of EA-FCCN is still superior to that of EA-FCN and EA-CCN catalysts. Moreover, in order to assess the intrinsic activity, we also calculated the mass activities and TOFs (Supplementary Fig. 24) on the basis of the total deposited metal amount. To be specific, EA-FCCN delivers a high mass activity of 3601 A g_metal_^−1^ at an overpotential of 300 mV, while 1183, 101, and 37 A g_metal_^−1^ are only observed for EA-FCN, EA-CCN, and RuO_2_, respectively (Fig. 2b). Similar tendency can also be observed for TOFs (Fig. 2b), where the TOF of EA-FCCN reaches 0.483 s^−1^ at an overpotential of 300 mV, 3.2, 34.5, and 41.1 times higher than that of EA-FCN, EA-CCN, and RuO_2_, respectively. Notably that the determined intrinsic activity values are inevitably underestimated, because only the surface metal species can endow veritably active sites rather than the whole deposited alloy film^24^. Such a remarkable OER activity of our as-obtained OER electrocatalysts (particularly for EA-FCCN) is superior to most of previously reported earth-abundant transition metal OER electrocatalysts (Supplementary Table 6).

To probe reaction kinetics, the Tafel plots of our catalysts were drawn, as depicted in Fig. 2c. The Tafel slope of EA-CCN is determined to be 51.4 mV  dec^−1^, which considerably decreases to 42.6 and 38.7 mV dec^−1^ for EA-FCN and EA-FCCN, respectively. The decreased Tafel slopes indicate the accelerated reaction kinetics and the probable change of rate-limiting step (RLS)^4,5^. Moreover, we measured the electrochemical impedance spectroscopy (EIS), where the smallest semicircle in Nyquist plot of EA-FCCN (Supplementary Fig. 25) indicates apparent decrease of interfacial charge transfer resistance (*R*_ct_), inducing the facilitated charge transfer to promote OER activity.

Stability was assessed through CV scanning and chronoamperometry tests. LSV polarization curve of EA-FCCN is well retained after 3000 CV scanning cycles (Fig. 2d), in which only an anodic shift of 8 mV is additional to reach a large current density of 400 mA cm^−2^. Besides, chronoamperometric curve of EA-FCCN (inset in Fig. 2d) displays a slightly decayed current density (decreasing only by ~7%) after 20-h continuous test, whereas RuO_2_ catalyst rapidly deactivates within 6 h, further corroborating the excellent stability. Structural characterizations (SEM and XPS, see Supplementary Figs. 26–27) after chronoamperometric test manifests that EA-FCCN is nearly remained without disturbing the bulk MCA film.

### Monitoring the dynamic evolution of metal sites during OER process

Our activity evaluations exhibit the superiority of Fe-involved electrocatalysts (especially for EA-FCCN) toward water oxidation. It is surmised that the interatomically electronic interplay in multimetal species plays a vital role on constructing highly active center to accelerate the reaction kinetics. Unfortunately, although unremitting efforts have been put, the catalytic mechanism is still ambiguous and elusive, especially for the critical role of Fe site, wherein some proposals are even conflicting. Many researchers proposed Ni site as catalytic center with Fe as a promoter. For instance, Görlin et al. reported that Fe^3+^ species diminished the charge contribution process of Ni^4+^ and stabilized Ni^2+^ to improve the catalytic activity^35^. Oppositely, Fe^3+^ species were also regarded as Lewis acid center to promote the formation of Ni^4+^ species^36,37^, inducing the exacerbated geometric distortions and electronic structural variation^38,39^. Meanwhile, high valence Fe^4+^ site was also detected by Mössbauer Spectroscopy to destabilize Ni^3+^ species in the NiOOH lattice^22^. Apart from Ni site as active center, attentions have also been put on whether Fe species can act as catalytic site. Evidences were offered by Friebel et al., in which Fe^3+^-O bond was shortened during catalytic process and Fe^3+^ site indeed offered a lower computational overpotential than Ni site^40^. This Fe catalytic center was also been experimentally strengthened by ^18^O-labbelled in situ Raman characterizations^41^. Moreover, investigations also demonstrated that Fe^3+^ could further evolve to high valence Fe^4+^ in Fe^4+^=O motif, contributing to the highly catalytic activity^42,43^. Very recently, Markovic and coworkers proposed a novel viewpoint that Fe site offered the high catalytic activity but must be preserved through the dynamic balance of Fe dissolution and redeposition on metal hydroxide surface^44^. Upon those complicated interpretations, it is of high importance for us to fundamentally understand the veritably catalytic sites and related reaction pathway in our catalytic system.

**Fig. 3:**
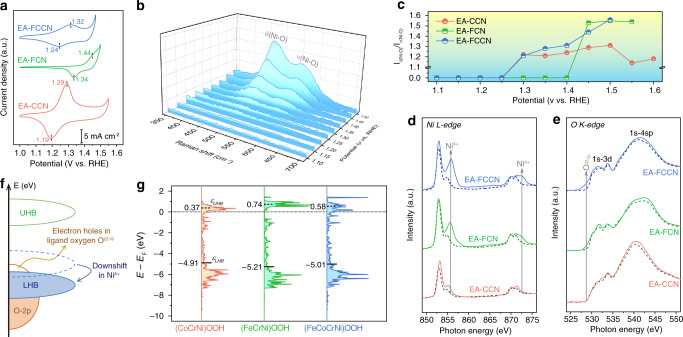
Identifications of ultimate active sites toward OER. **a** Steady CV scanning curves of electrochemical activated MCA films. **b** Electrochemical in situ Raman spectra of EA-FCCN at the range of 350–700 cm^−1^ at the operated potentials from 1.1 to 1.5 V versus RHE. **c***δ*(Ni-O)-to-*ν*(Ni-O) ratios in electrochemical in situ Raman spectra of various catalysts related to the operated potentials. **d**, **e** Ex situ sXAS measurements of various catalysts. **d** Ni *L*-edge and (**e**) O *K*-edge sXAS spectra. Dash and solid lines represent the spectrums collected at open circuit condition and the operated potentials (i.e., 1.5 V for EA-FCCN, 1.55 V for EA-FCN, and 1.6 V for EA-CCN), respectively. **f** Schematic representation of activating oxygen ligands induced by Ni^4+^ species in Mott-Hubbard model. **g** Computational 3*d*-orbitals PDOS diagrams of localized Ni site adjacent to Fe/Co in various oxyhydroxide models, together with the related band centers.

Given the dynamic surface reconstruction of catalysts prior to water oxidation, the redox electrochemistry of metal species in various catalysts are investigated, which is supposed to be decisive for constructing active sites^11,21^. As displayed in steady CV scanning curves (Fig. 3a), the anodic oxidation peaks can be ascribed to by Ni^2+^→Ni^3/4+^ oxidation along with hydroxyl deprotonation^23,45,46^. Two facts can support our conjecture: (1) Ni^2+^, Co^3+^, and Fe^3+^ species predominantly exist on the surface of Cr-leached MO_*x*_H_*y*_ (evidenced by XPS and sXAS, vide supra); and (2) oxidation of Co^3+^ and Fe^3+^ species typically requires much more elevated potentials^21,22^. The oxidation of Ni^2+^ species signifies further surface evolution into oxyhydroxides to offer the veritably active sites for OER^19,47^. The varied oxidation peaks for different catalysts imply their divergent evolution of Ni^2+^ species. The peak for EA-FCN (1.44 V (versus RHE)) positively shifts compared to that of EA-CCN (1.29 V) and EA-FCCN (1.32 V), indicating that Ni^2+^ oxidation is largely delayed by Fe^3+^ species in EA-FCN^22,23,46^. To understand the surface evolution, we monitored the dynamic changes of out catalysts using electrochemical in situ Raman spectroscopy. Two characteristic Raman signals of Ni^3+^-O at 472 and 554 cm^−1^ can be gradually recognized on the recorded spectrums with the elevated potentials (Fig. 3b and Supplementary Fig. 28), which can be indexed to the *E*_g_ bending vibration (*δ*(Ni-O)) and *A*_1g_ stretching vibration (*ν*(Ni-O)) mode in nickel oxyhydroxide (NiOOH), respectively^46,47^. The emergence of Raman signals is well associated with the anodic oxidation peaks in positive CV scanning curves (Supplementary Fig. 29), reaffirming the dynamic surface reconstruction into oxyhydroxides. Notably, the variation of *δ*(Ni-O)-to-*ν*(Ni-O) ratios (labelled to *I*_δ/ν_) are obviously different for our catalysts (Fig. 3c), signifying the distinguishing lattice structure of formed NiOOH. Generally, NiOOH contains *β* and *γ* phases, where the intensity of *ν*(Ni-O) is relatively lower (i.e., higher *I*_δ/ν_) in *γ*-NiOOH due to its looser structure with more disorder^45,47^. As observed in Fig. 3c, the initial *I*_δ/ν_ values for EA-CCN and EA-FCCN are about 1.2, while it aberrantly reaches 1.55 for EA-FCN. Such a higher *I*_δ/ν_ for EA-FCN manifests the direct formation of *γ*-NiOOH structure when surface oxidation occurs^45,47^. It is regarded that highly oxidized Ni^4+^ species exists in *γ*-NiOOH due to the statistic Ni valency of +3.6^36,48^. Therefore, we infer that Ni^2+^ species can be directly oxidized to Ni^4+^ species in EA-FCN, while normal Ni^3+^ species is initially formed in EA-CCN and EA-FCCN. Based on the Pourbaix diagrams, the deep Ni^2+^→Ni^4+^ oxidation is energetically unfavorable^15,16^, which interprets the anodically shifted oxidation peak of EA-FCN in CV scanning curve (Fig. 3a). Moreover, we speculate that the direct Ni^4+^ formation is accomplished by assistance of Fe^3+^ species due to the robust PET effect in Fe-O-Ni moiety, which efficiently withdraws the electrons from Ni to Fe, stabilizing Ni^4+^ species (see “Discussions” in Supplementary Note 3)^22,38^. Given that highly oxidized Ni^4+^ species offers higher intrinsic activity^36–39^, only a little potential barrier of 45 mV is applied for EA-FCN to reach a current density of 10 mA cm^−1^ after Ni^2+^ oxidation (Supplementary Fig. 30). Contrarily, a much larger potential barrier (344 mV) is essential for EA-CCN with only Ni^3+^ species during OER process. Different from EA-CCN, profited from the existence of Fe^3+^ species, *γ*-NiOOH structure with Ni^4+^ species is also constructed in EA-FCCN when the potential is further elevated, as monitored by in situ Raman spectra (Fig. 3c, *I*_δ/ν_ = 1.56 at potential of 1.5 V). Therewith, a substantially reduced potential barrier (131 mV, Supplementary Fig. 30) is required for EA-FCCN to initiate OER. In view of the initially formed *β*-NiOOH structure with only Ni^3+^ species, we proposed a multistep evolution of Ni species (i.e., Ni^2+^→Ni^3+^→Ni^4+^) in EA-FCCN during OER process, which can bypass the energy obstacle of direct Ni^4+^ formation, lowering the overall potential barrier to initiate OER.

The ultimate electronic states of metal species at OER conditions were also substantiated by ex situ sXAS after treatment at different operated potentials^10,16,33^. As disclosed in Co and Fe *L*-edge sXAS spectra (Supplementary Fig. 31), Co^3+^ and Fe^3+^ species are well maintained without further oxidation at OER conditions. However, Ni species undergoes distinctive evolution (Fig. 3d). Additional white line (blue-shift of ~2.8 eV against the main peak of Ni^2+^ species) can be recognized in Ni *L*-edge sXAS spectra for EA-FCN and EA-FCCN at OER conditions, which can be ascribed to dynamically formed Ni^4+^ species^10,16^, in line with electrochemical in situ Raman measurements. Considering the correlation between structural evolution and OER activity, we conclude that the dynamically formed Ni^4+^ species in EA-FCN and EA-FCCN serves as veritably active sites for (at least plays a decisive role on) the OER.

Upon the distinctive Ni states in different catalysts during catalytic process, one can envision that the electronic structure should be largely steered^25,39^. For late transition metals, *d-*orbitals can be further split into electron-filled lower Hubbard band (LHB) and empty upper Hubbard band (UHB) owing to strong *d-d* on-site Coulomb interaction (*U*), namely Mott-Hubbard splitting^12,49^. Zaanen-Sawatzky-Allen scheme depicts that the increased valence of metal cation with orbital volume shrinkage (e.g., highly oxidized Ni^4+^ species) can expand *U* to exacerbate this splitting^36,50^. Thus, LHB will downshifts to probably penetrate *p*-band of oxygen ligands as schemed in Fig. 3f, exhibiting charge-transfer insulator character. This modulation of electronic structure can be visualized by the computational 3*d*-orbtial partial density of states (PDOS) diagrams of localized Ni sites adjacent to Fe/Co in our established metal oxyhydroxide models (labelled to MOOH, see Supplementary Fig. 32 and Supplementary Note 4)^48,51^. As plotted in Fig. 3g, the computed *d*-band centers (*ε*_d_) apparently downshifts for Fe-involved (FeCrNi)OOH and (FeCoCrNi)OOH models, far away from the Fermi level (*E*_F_). As the PDOS below *E*_F_ describes the occupied 3*d*-orbitals distribution, we can regard the related *ε*_d_ as LHB-band center (*ε*_LHB_). Similarly, UHB-band center (*ε*_UHB_) can also be calculated from PDOS diagrams based on the orbital distribution above *E*_F_ (Fig. 3g). Here we qualitatively determine *U* as the energy difference between *ε*_LHB_ and *ε*_UHB_ (i.e., *δ*(*ε*_UHB_ − *ε*_LHB_)), showing that Fe-involved models deliver the enlarged *U* values (Supplementary Fig. 33). We ascribe the enlarged *U* along with downshifted LHB to the dynamically formed high valence Ni^4+^ species, as experimentally evidenced by Raman and sXAS measurements.

To deeply investigate the influence of this altered electronic structure, the evolution of surface oxygen species was monitored by ex situ O *K*-edge sXAS spectra. Figure 3e exhibits the shoulder adsorption (located at ~529 eV) prior to the pre-edge absorption for EA-FCN and EA-FCCN at OER conditions. This meaningful signal can be assigned to activated oxygen ligands containing localized holes (i.e., O^(2−δ)−^)^52,53^. Its concomitant emergence with Ni^4+^ species manifests that the formed O^(2−δ)−^ species is derived from the downshifted 3*d*-band of Ni^4+^ species, just as predicted by computational PDOS diagrams. Therefore, oxygen ligands can thermodynamically lose the electrons from *p*-band, leaving holes to destabilize and activate themselves, as schemed in Fig. 3f. The localized holes enable oxygen ligands electrophilic and chemically active to participate in water oxidation through LOM pathway. Moreover, it can also be observed that O *K*-edge sXAS spectra exhibit the increased intensity at OER conditions for all the catalysts (Fig. 3e), indicating the enhanced metal–oxygen covalency to promote the charge transfer between metal center and oxygen atoms during OER process^9,39^.

### In-depth understanding of reaction pathway

**Fig. 4:**
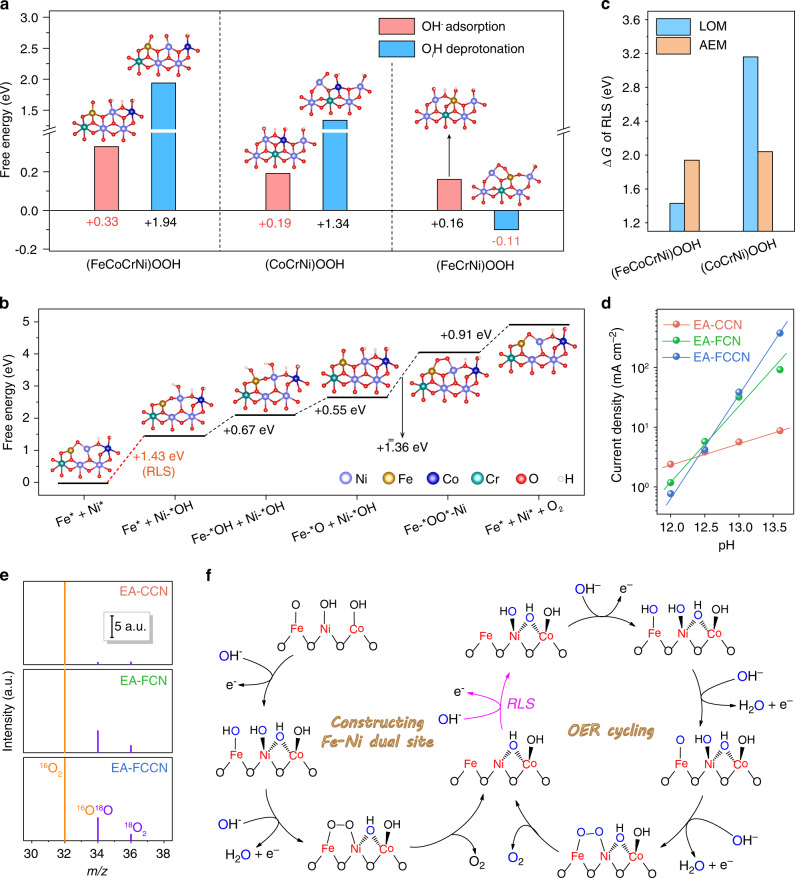
Investigations of proposed OER pathway. **a** Free energy comparation between OH^−^ adsorption and lattice hydroxyl deprotonation in different models. **b** Free energy diagram of OER cycling at Fe–Ni dual-site on (FeCoCrNi)OOH model. **c** The determined Δ*G* of RLS via LOM and AEM pathway in different models. **d** The determined current densities of various catalysts at 1.5 V versus RHE under different pH values. **e** The detected MS signals of generated oxygen molecule using ^18^O isotope-labelled catalysts. The signals are normalized through initializing the intensity of ^16^O_2_ as 1000 a.u. **f** Schematic illustration of the proposed overall OER pathway for EA-FCCN catalyst. The black and blue oxygen atoms represent lattice and adsorbed oxygen, respectively.

As mentioned above, the highly oxidized Ni^4+^ species is dynamically formed and activates the lattice oxygen ligands with electron holes, acting as a crucial knob on promoting OER activity. To shed more light on catalytic nature, density functional theory (DFT) simulations were conducted to systematically screen the evolution of surface oxygen species on different oxyhydroxide models, considering both LOM and adsorbate evolution mechanism (AEM) pathways (see computational details in Supplementary Note 5)^12,18^. The lattice and adsorbed oxygen atoms in the models are marked to O_*l*_ and O_*a*_, respectively. For (CoCrNi)OOH and (FeCoCrNi)OOH models, OH^−^ adsorption (namely, forming Ni-*μ*-O_*a*_H-Co motif) is more energetically preferential than deprotonation of Ni-terminated hydroxyl (Ni-*η*-O_*l*_H), while (FeCrNi)OOH model exhibit inverse scene (Fig. 4a). Moreover, the negative Gibbs free energy difference (Δ*G*) of Ni-*η*-O_*l*_H deprotonation on (FeCrNi)OOH model (−0.10 eV) means its spontaneous nature, indicating the further evolution of Ni site to Ni^4+^ species. Meanwhiles, the deprotonated (FeCrNi)OOH model after structural relaxation (inset in Fig. 4a) contains an Fe-(*μ*-O_*l*_O_*l*_)-Ni motif with O–O bond length of 1.39 Å (Supplementary Fig. 34b). The formed peroxo-like *μ*-O_*l*_O_*l*_ species is inferred to derive from intramolecularly nucleophilic coupling of deprotonated *η*-O_*l*_ atoms, which contains ligand holes introduced by highly oxidized Ni^4+^ species, as proofed by O *K*-edge sXAS (Fig. 3e). This evolution manifests that oxygen evolution proceeds with a direct Mars-van Krevelen-like mechanism on (FeCrNi)OOH model, undergoing LOM pathway through desorbing *μ*-O_*l*_O_*l*_ species (Supplementary Fig. 35a, left). Meanwhile, this Fe-(*μ*-O_*l*_O_*l*_)-Ni motif also indicates Fe–Ni dual-site as ultimate catalytic center for oxygen evolution (see Supplementary Fig. 36 and Supplementary Note 6). Afterwards, OH^−^ anions will refill the oxygen vacancies at Fe–Ni dual-site and loop the deprotonation-coupling-desorption process (Supplementary Fig. 35a, right). The simulated energy barrier diagram of OER cycling (Supplementary Fig. 35b) exhibits that RLS is the deprotonation of Fe-*η*-OH with an energy uphill of 1.54 eV.

Now we turn to the oxygen evolution on (FeCoCrNi)OOH and (CoCrNi)OOH models after OH^−^ pre-adsorption. Computational results demonstrate that intramolecular LOC can also occurs on (FeCoCrNi)OOH model after Ni-*η*-O_*l*_H deprotonation (Δ*G* = 1.36 eV, see Supplementary Fig. 37), forming *μ*-O_*l*_O_*l*_ species with bond length of 1.41 Å (see Supplementary Fig. 34a). The successful LOC indicates that (FeCoCrNi)OOH model also evolves oxygen molecule via LOM pathway, analogous to (FeCrNi)OOH model. Peroxo-like oxygen species is experimentally discerned by electrochemical in situ Raman spectra (Supplementary Fig. 38). The broad signals at the range of 850–1150 cm^−1^ can be assignable to active oxygen species in Ni(OO)^–^ species^54,55^, whose emergence in EA-FCN and EA-FCCN strengthens the preferential step of intramolecular LOC. Then, we simulated the OER cycling at Fe–Ni dual-site and determined the first OH^−^ adsorption as RLS with an energy barrier of 1.43 eV for (FeCoCrNi)OOH model (Fig. 4b). The lower energy barrier related to (FeCrNi)OOH model predicts the more superior OER activity of EA-FCCN catalyst, coinciding with the experimental results. The bridging pre-adsorbed OH^−^ at Ni-Co site does not directly involve OER process but acts as an electron-withdrawing modifier to favor hydroxyl deprotonation^56,57^, thereby reducing the overall barrier of OER cycling. Conventional AEM pathway is also considered through direct deprotonation of adsorbed *μ*-O_*a*_H, delivering an energy barrier as high as 1.94 eV (Supplementary Fig. 39). The computed energy barrier manifests that LOM pathway is more energetically favorable on (FeCoCrNi)OOH model (Fig. 4c). In terms of (CoCrNi)OOH model, inverse result is observed that AEM pathway hold a lower energy barrier than LOM pathway (Fig. 4c and Supplementary Fig. 40). In comparison of Fe-involved model, (CoCrNi)OOH model requires a more elevated energy uphill to furnish the OER cycling, foreboding the lower intrinsic activity of EA-CCN catalyst. We speculate that the oxygen atoms can hardly be activated by the ligand holes without the high valence Ni^4+^ species, leading to the unfavorable O–O bond formation via OH^−^ nucleophilic attack (i.e., RLS) for (CoCrNi)OOH model. Taken together, we conclude that oxygen molecule evolves via LOM pathway for EA-FCN and EA-FCCN catalysts and AEM pathway for EA-CCN pathway, respectively. Moreover, it should also be noted that Fe-involved models show the much looser structure than (CoCrNi)OOH model during OER cycling (reflected by metal-metal bond length variation in Supplementary Fig. 41), indicating the geometric distortion near Fe site. This distortion was reported to favor Ni species evolution, thereby improving the catalytic activity^38^.

The proposed free energy diagrams manifest that O–O coupling is significantly facilitated and no longer limits the reaction for LOM pathway in Fe-involved models. As known, LOM pathway typically involves the non-concerted proton-electron transfer step of RLS (Supplementary Fig. 42), originating from the mismatch of electron transfer kinetics and hydroxide affinity at the oxide/electrolyte interface (see details in Supplementary Note 7)^12,18^. Thus, proton-electron transfer is decoupled in RLS, exhibiting a pH-dependent activity. As expected, our Fe-involved catalysts shows much more sensitive correlation between pH and OER activity (Fig. 4d and Supplementary Fig. 43), strengthening the argument of LOM pathway. To further clarify the oxidation of lattice oxygen ligands, an ^18^O isotope-labelled experiment was designed^18,20^, as schemed in Supplementary Fig. 44. When ^18^O-labelled catalysts with M^18^O_*x*_H_*y*_ surface (Supplementary Fig. 45) are used to carry out OER, EA-FCCN and EA-FCN catalysts generate ^18^O-labelled products (i.e., ^16^O^18^O and ^18^O_2_ molecule) while EA-CCN cannot, as detected by MS (Fig. 4e). This straightforward evidence unambiguously corroborates the participation of lattice oxygen ligands into oxygen evolution for our Fe-involved catalysts.

By combining experimental evidences with simulated results, we propose an overall illustration towards OER on (FeCoCrNi)OOH model to rationalize the enhanced OER activity of corresponding EA-FCCN catalyst, as depicted in Fig. 4f. The interatomically electron interplay plays an organizing knob throughout the whole process on optimization of the reaction pathway. Ni^4+^ species are favorably formed through a multistep evolution (Ni^2+^→Ni^3+^→Ni^4+^) induced by the electronic modulation through Fe-O-Ni moiety and pre-adsorbed *μ*-O_*a*_H species at Ni-Co site, introducing holes into oxygen ligands to evoke LOM pathway via Mars-van Krevelen-like mechanism, then driving the construction of Fe–Ni dual-site as ultimate catalytic center to loop the water oxidation. As a result, benefiting from the favorable formation of Ni^4+^ species and dynamically constructed Fe–Ni dual-site, EA-FCCN catalyst offers low overpotential and superb activity for OER.

## Discussion

In summary, we have demonstrated an FeCoCrNi MCA film as highly efficient OER electrocatalyst after irreversible surface reconstruction into Cr-leached multimetal (oxy)hydroxides. High oxidized Ni^4+^ species is dynamically formed and subtly engineered by the interatomically electronic interplay, playing a decisive role on constructing ultimate catalytic center, as fundamentally understood through electrochemical voltammetry, in situ Raman spectroscopy and ex situ sXAS techniques, as well as theoretical simulations. Fe component is unveiled to induce electron depletion in Ni species to ensure the formation of Ni^4+^ species. Meanwhile, the high energy barrier of Ni^4+^ formation is alleviated by a multistep evolution of Ni^2+^→Ni^3+^→Ni^4+^, which is facilitated by pre-absorbed hydroxyl at Ni-Co site. The formed Ni^4+^ species enable oxygen ligands electrophilic through introducing holes, favoring intramolecular LOC to evoke LOM pathway via Mars-van Krevelen-like mechanism, corroborated by ^18^O isotope-labelled experiments. An Fe–Ni dual-site is proposed to be dynamically constructed as ultimate catalytic center, achieving high OER activity and accelerating the reaction kinetics. As a result, the surface reconstructed FeCoCrNi MCA film electrocatalyst delivers the excellent mass activity and turnover frequency (TOF) of 3601 A g_metal_^−1^ and 0.483 s^−1^ at an overpotential of 300 mV in alkaline electrolyte, respectively. This work endows a guideline for the exploration of advanced OER electrocatalysts as well as broadens the applications of multicomponent alloy materials toward catalysis.

## Methods

### Chemicals

High purity alloy targets (>99.99%) were customized from Beijing JAH TECH Co., Ltd, which were prepared by metallurgy using high purity (>99.99%) metals (nickel, cobalt, iron, and chromium) as raw materials. Ruthenium oxide (RuO_2_, 99.95%) was purchased from Adamas-beta. ^18^O isotope-labelled H_2_^18^O (97 atom% of ^18^O) was purchased from J&K. Carbon black (VXC-72R) was obtained from CABOT. Carbon cloth (CC, W0S1009) was purchased from CeTech Co., Ltd. Other chemicals were obtained from Aladdin. The water used in all experiments was de-ionized (DI).

### Deposition of MCA films on CCs

A series of nominally equiatomic MCA films (i.e., FeCoCrNi, FeCrNi, and CoCrNi) with the thickness of about 100 nm were deposited on CC substrates from the related alloy targets through a magnetron sputtering method at room temperature^29,30^. Prior to sputtering, the targets were initially cleaned by Ar^+^ bombardment for 2 min to remove the surface oxide and possible contaminants. CC substrates were ultrasonically cleaned using acetone, ethanol, and de-ionized water for 30 min, respectively. High purity argon was introduced into the vacuum chamber once the base pressure was below 5.0 × 10^−4^ Pa. The total flow of argon flow rate was fixed at 12 sccm (standard cubic centimeters per minute) and the rotation speed was 10 rpm (revolutions per minute) to homogenize the alloy composition and film thickness. The substrates were neither cooled nor heated during deposition. The thickness of MCA films was adjusted by the sputtering time while other parameters were kept constant.

### Electrochemical activation of MCA films

Surface activation of MCA films coated on CCs were performed by the electrochemical CV scanning in a standard three-electrode electrochemical cell using 1-M KOH solution as electrolyte. The MCA film samples, platinum plate and Hg/HgO (1-M KOH) served as working electrode, counting electrode and reference electrode, respectively. In all, 200 cycles of CV scanning were conducted for each sample in the potential region from 0 to 0.6 V versus Hg/HgO at a sweep rate of 100 mV s^−1^ to activate the surface. The electrochemical activated FeCoCrNi, FeCrNi and CoCrNi MCA films were simplified as EA-FCCN, EA-FCN, and EA-CCN, respectively.

### Material characterizations

SEM and EDS images were taken on a JEOL JSM-6490 field emission scanning electron microscope operated at 5 kV with EDS detector. Transmission electron microscope (TEM), high-resolution TEM (HRTEM), and selected area electron diffraction (SAED) images were collected on a JEOL JEM-2100F field-emission high-resolution transmission electron microscope operated at 200 kV. The samples were prepared through ion-milling at the temperature of 223 K. 3D atomic-probe tomography (APT) characterizations were performed in a CAMEACA LEAP 5000 XR local electrode atom probe. The specimens were analyzed at 45 K in laser mode at a laser energy of 100 pJ, pulse rate of 200 kHz, and detection rate of 0.5%. Imago Visualization and Analysis Software (IVAS) version 3.8 was used for creating the 3D reconstructions and conducting the data analysis. X-ray diffraction (XRD) patterns were recorded by using a Rigaku SmartLab X-ray diffractometer with Cu-Kα radiation (*λ* = 1.5418 Å). The loading mass of samples on CC were measured with a Thermo Scientific Plasma Quad 3 inductively coupled plasma mass spectrometry (ICP-MS) after dissolving the samples with aqua regia solution. Each sample was measured for three times to minimize the error. XPS spectra were collected on a Thermo Scientific Escalab 250Xi X-ray photoelectron spectrometer, using non-monochromatized Al-Kα X-ray (1486.6 eV) as the excitation source.

### Electrochemical measurements

All of the electrochemical measurements for OER were performed using a CHI 660D electrochemical workstation (Shanghai Chenhua, China) in a standard three-electrode electrochemical cell with O_2_-saturated 1-M KOH solution as electrolyte^58^. The as-prepared MCA/CC samples (1 × 1 cm^2^) act as working electrode. Commercial RuO_2_ powder mixing with conductive carbon black (20 wt%) was drop-coated on a pretreated CC with the loading mass of 0.5 mg cm^−2^, serving as the benchmark catalyst. A platinum plate (1 × 1 cm^2^) and Hg/HgO (1-M KOH) served as counting electrode and reference electrode, respectively. The measured potentials versus Hg/HgO could be converted to the potentials versus reversible hydrogen electrode (RHE) by Equation (1):

1 $$E_{{\mathrm{RHE}}} = E_{{\mathrm{Hg/HgO}}} + 0.098 + 0.059 \times {\mathrm{pH}}$$

Prior to the assessment of electrochemical performance, several CV scanning cycles were performed to stabilize the catalysts. LSV polarization curves were measured at a sweep rate of 5 mV s^−1^, and the potentials were corrected by 95%-*iR* compensation to eliminate the effect of solution resistance^59^. The Tafel plots were obtained by the corresponding LSV polarization curves plotted as overpotential versus the log current (log[*j*]). The stability tests were performed by chronoamperometry for 20 h at the initial current density of ~10 mA cm^−2^ and CV scanning for 3000 cycles in the potential region from 0.2 to 0.7 V versus Hg/HgO at a sweep rate of 100 mV s^−1^. *C*_dl_ was determined from the CV scanning curves measured in the non-Faradaic potential range from 0.85 to 1 V versus RHE. The sweep rates were set to be 2, 5, 10, 20, 30, 50 mV s^−1^, respectively. *C*_dl_ was estimated by plotting the Δ*J* = (*J*_+_ − *J*_−_)/2 at 0.925 V versus RHE against the sweep rates. ECSA was calculated by Equation (2):

2 $${\mathrm{ECSA}} = C_{{\mathrm{dl}}}/C_{\mathrm{s}} \times A$$

where *C*_s_ is the capacitance of an atomically smooth planar surface (0.04 mF  cm^−2^ in alkaline media^60^), and *A* is the electrode area (1 cm^−2^ for our working electrodes). EIS were carried out in ZAHNER Electrochemical workstation with the frequency ranging from 10^−2^ to 10^5^ Hz with an amplitude of 5 mV. To assess the intrinsic activity of our as-obtained samples, both mass activities and TOFs were calculated by Eqs. (3) and (4):

3 $${\mathrm{mass}}\,{\mathrm{activity}} = \left( {j \times A} \right)/m$$

4 $${\mathrm{TOF}} = \left( {j \times A} \right)/\left( {4 \times F \times n} \right)$$

where *j* was the current density, *A* was the geometric area of electrode, *F* was the Faraday constant (96,485 C mol^−1^), *m* was the loading mass of sample, and *n* was the number of active sites. For our assessment, the loading mass of samples was determined by ICP-MS, and all the deposited metal atoms were considered to be the active sites. To performed ^18^O isotope-labelled experiments, MCA films were firstly activated in ^18^O-labelled Na^18^OH electrolyte to form M^18^O_*x*_H_*y*_ surface (schemed in Supplementary Fig. 43). Then, OER measurements were conducted in a sealed electrochemical cell with N_2_-saturated K^16^OH electrolyte at a current density of *ca*. 10 mA cm^−2^ for 2 min. Afterwards, the gas product was extracted and analyzed using gas chromatography-mass spectrometry (GC-MS, 7890A and 5975C, Agilent).

### Electrochemical in situ Raman spectra measurements

Electrochemical in situ Raman spectra were recorded in the WITEC alpha300R confocal Raman imaging equipment using a 633-nm laser with the power of 17 mW. The Raman frequencies were corrected using silicon wafer. A home-built top-plate electrochemical cell was used for in situ Raman spectra measurements, in which a platinum plate and Ag/AgCl (saturated KCl) served as counting electrode and reference electrode, respectively. O_2_-saturated 1-M KOH solution was act as electrolyte to inject into the cell. To monitor the evolution of catalyst samples during OER process, Raman spectrum was collected after a constant potential was applied to the catalyst electrode for 10 min. Each Raman spectrum was obtained by the integration time of 10 s with accumulating 5 times.

### Ex situ sXAS measurements

Ex situ metal (Ni, Co, and Fe) *L*-edge and O *K*-edge sXAS measurements after treatment at different operated potentials were performed at the beamline BL12B-a of National Synchrotron Radiation Laboratory (NSRL) in Hefei, China. The electron beam energy of the storage ring was 800 MeV with an average stored current of 300 mA. A bending magnet was connected to the beamline and equipped with three gratings covering photon energies from 100 to 1000 eV with an energy resolution of ~0.2 eV. All of data were recorded in the TEY mode by collecting the sample drain current under a vacuum greater than 5 × 10^−8^ Pa. The resolving power of the grating was typically *E*/∆*E* = 1000, and the photon flux was 5 × 10^8^ photons per second. The catalyst samples were pretreated under different operated potentials for 10 min, in which the operated potentials were desired to ensure the proceeding of OER (i.e., 1.5, 1.55 and 1.6 V versus RHE for EA-FCCN, EA-FCN and EA-CCN, respectively). Afterwards, the as-treated samples were freeze-quenched by liquid N_2_ and stored in liquid N_2_ before sXAS measurements to minimize the surface degradation^15,21^. For sXAS tests, the as-treated samples were taken out from liquid N_2_ and quickly put into a high vacuum chamber. The related sXAS spectra were collected at room temperature.

### Theoretical simulations

DFT simulations were performed with the Vienna ab initio simulation package (VASP)^61,62^. The generalized gradient approximation (GGA) of the Perdew-Burke-Ernzerhof (PBE) functional^63^ and the projector augmented-wave (PAW) potential^64^ were employed. Grimme method^65^ was used to consider the weak van der Waals’ for layer materials. *β*-NiOOH with (10 − 14)-terminated surface was selected as our pristine slab model^51^, and 4 × 4 supercell was established. The periodic boundary condition (PBC) was set with a 20 Å vacuum region above surface to avoid the attractions from adjacent periodic mirror images. To simplify our calculations, other metal atoms (i.e., Fe, Co, and Cr) were introduced into the slab model to substitute for (sub)surface Ni atoms, simulating the catalytic sites of multimetal (oxy)hydroxides structure formed during OER process (see Supplementary Note 4). An energy cutoff of 500 eV was used for the plane-wave expansion of the electronic wave function for all numerical calculations with a Monkhorst-Pack mesh of 5 × 5 × 1. The force and energy convergence criterion were set to be 10^−2^ eV Å^−1^ and 10^−5^ eV, respectively. The relative location of various metal atoms was determined by the slab model with the lowest energy.

The computational hydrogen electrode (CHE) model^66–68^ was applied to simulate the OER pathway and determine the reaction energy barrier for different slab models. Various oxygen species intermediates were considered and the related adsorption energy (Δ*E*_ads_) of those intermediates were calculated according to Eq. (5):

5 $$\Delta E_{{\mathrm{ads}}} = E_{{\mathrm{total}}}-E_{{\mathrm{sub}}}-E_{{\mathrm{ads}}}$$

where *E*_total_, *E*_sub_, and *E*_ads_ represent the total energies of the systems, the substrates, and the adsorbates, respectively. The Gibbs free energy change (Δ*G*) of each step was defined as Eq. (6):

6 $$\Delta G = \Delta E + \Delta ZPE--T\Delta S$$

where Δ*E* is the electronic energy difference, Δ*ZPE* and Δ*S* are the difference of zero-point energies and the change of entropy, respectively, which were estimated from the vibrational frequencies, and *T* = 298.15 K. The computational details were shown in Supplementary Note 5.

## Supplementary information

Supplementary Information

## Data Availability

The data supporting the findings of this study are available from the corresponding author upon reasonable request.
