# Supplementary material for: Lattice oxygen activation enabled by high-valence metal sites for enhanced water oxidation
Source: Nat Commun. 2020 Aug 13;11:4066. doi: 10.1038/s41467-020-17934-7 (PMC7426847; doi:10.1038/s41467-020-17934-7)
Supplement: Supplementary file 1 — Supplementary Information [file 41467_2020_17934_MOESM1_ESM.pdf]

SUPPLEMENTARY INFORMATION

Lattice oxygen activation enabled by high-valence metal sites for enhanced water oxidation

Ning Zhang[1,4 ‖], Xiaobin Feng[2,6, ‖], Dewei Rao[3, ‖], Xi Deng[5], Lejuan Cai[1,4], Bocheng Qiu[1,4], Ran Long[5], Yujie Xiong[5], Yang Lu[2,6]* and Yang Chai[1,4]*

[1]Department of Applied Physics, The Hong Kong Polytechnic University, Hung Hom, Kowloon, Hong Kong, P. R. China

[2]Department of Mechanical Engineering, City University of Hong Kong, Kowloon, Hong Kong, P. R. China

[3]School of Materials Science and Engineering, Jiangsu University, Zhenjiang, Jiangsu 212013, P. R. China

[4]The Hong Kong Polytechnic University Shenzhen Research Institute, Shenzhen 518057, P. R. China

[5]Hefei National Laboratory for Physical Sciences at the Microscale, iChEM (Collaborative Innovation Center of Chemistry for Energy Materials), School of Chemistry and Materials Science, National Synchrotron Radiation Laboratory, University of Science and Technology of China, Hefei, Anhui 230026, P. R. China

[6]Nano-Manufacturing Laboratory (NML), Shenzhen Research Institute of City University of Hong Kong, Shenzhen 518057, P. R. China

‖These authors contributed equally: Ning Zhang, Xiaobin Feng and Dewei Rao

*Corresponding author: E-mail: yanglu@cityu.edu.hk (Y.L.); ychai@polyu.edu.hk (Y.C.)

Supplementary figures.

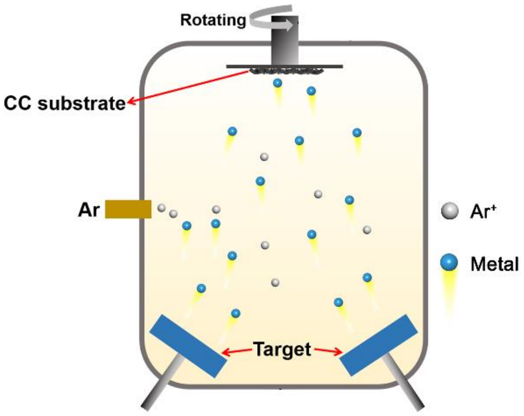

Supplementary figure 1 | Schematic illustration of magnetron sputtering technique.

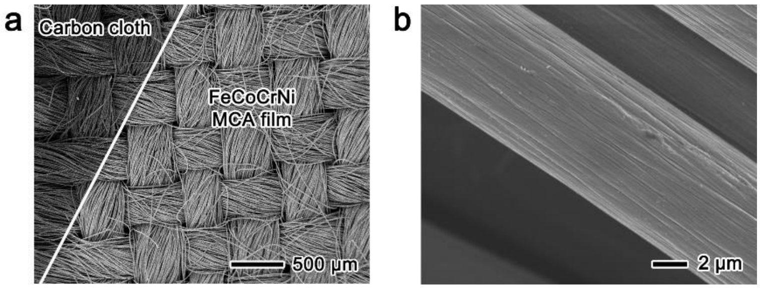

Supplementary figure 2 | Additional SEM images of FeCoCrNi MCA film coated on CC. (**a**) Low magnification. (**b**) high magnification.

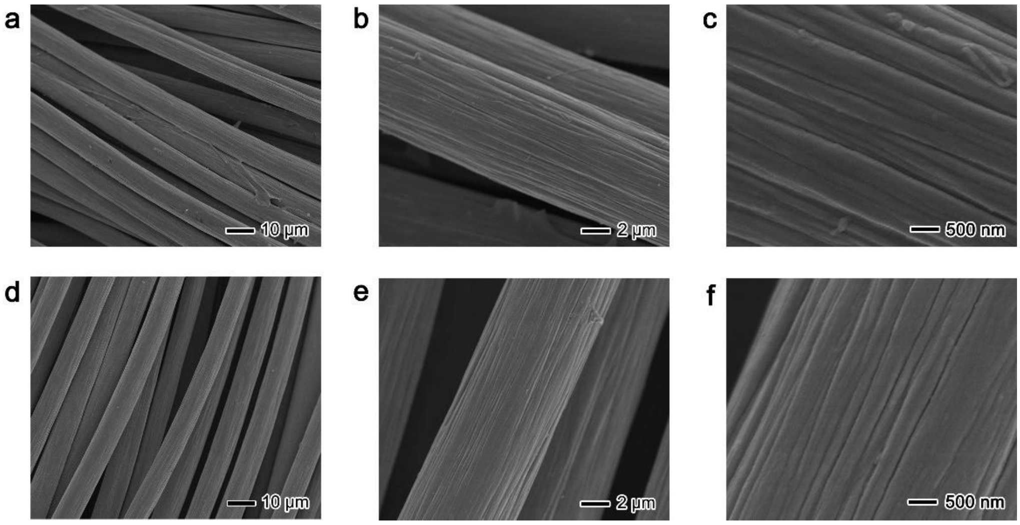

Supplementary figure 3 | SEM images of other MCA films coated on CCs. (**a-c**) FeCrNi. (**d-f**) CoCrNi.

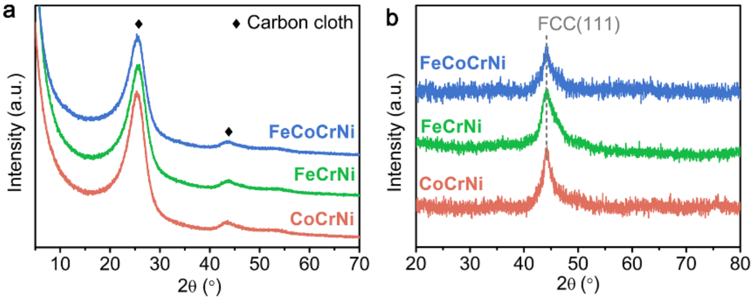

Supplementary figure 4 | XRD patterns of MCA films. The MCA samples are coated on (**a**) CCs and (**b**) Si wafers, respectively. The XRD peaks of MCA films coated on CCs cannot be recognized, probably due to low diffraction effect induced by the 3D spatial structure of CC. To analyze the phase of MCA films, Si wafers were employed as substrates to deposit the MCA films using the same procedure.

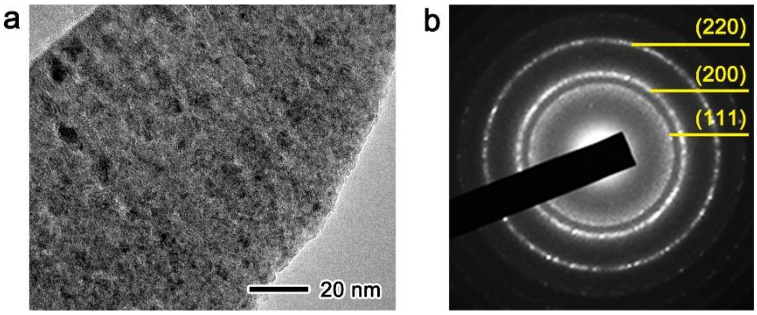

Supplementary figure 5 | TEM characterization of FeCoCrNi MCA sample. (**a**) TEM image. (**b**) The corresponding selected area electron diffraction (SAED) pattern. The sample was ion-milled at a temperature of 223 K.

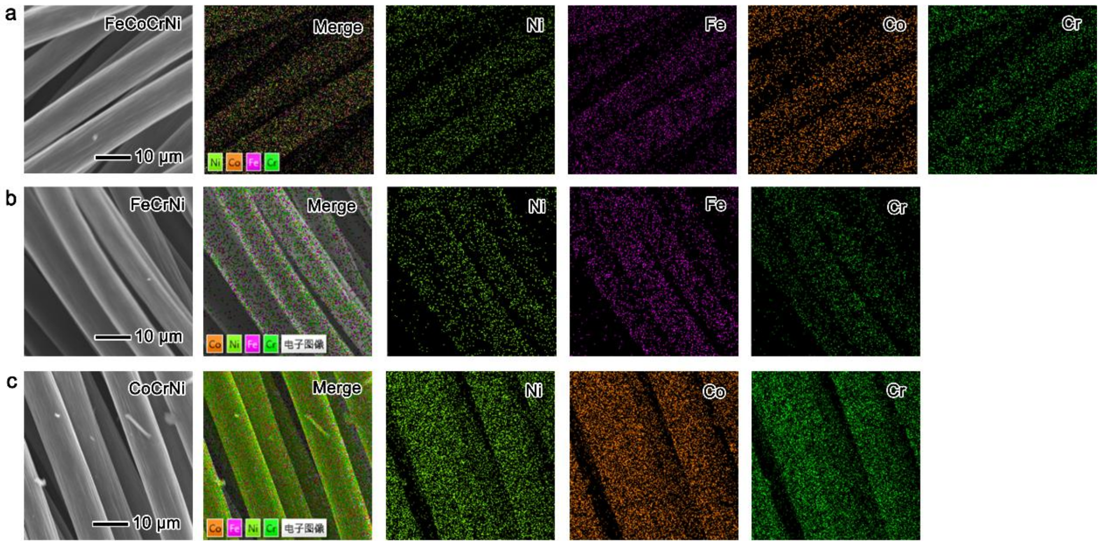

Supplementary figure 6 | SEM-EDS elemental mapping images of MCA films coated on CCs. (**a**) FeCoCrNi. (**b**) FeCrNi. (**c**) CoCrNi.

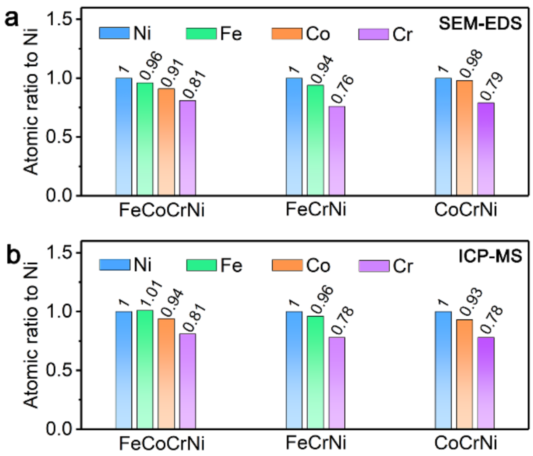

Supplementary figure 7 | The determined atomic ratios of the elements in MCA films. (**a**) SEM-EDS results. (**b**) ICP-MS results. The atomic ratios were normalized by Ni component.

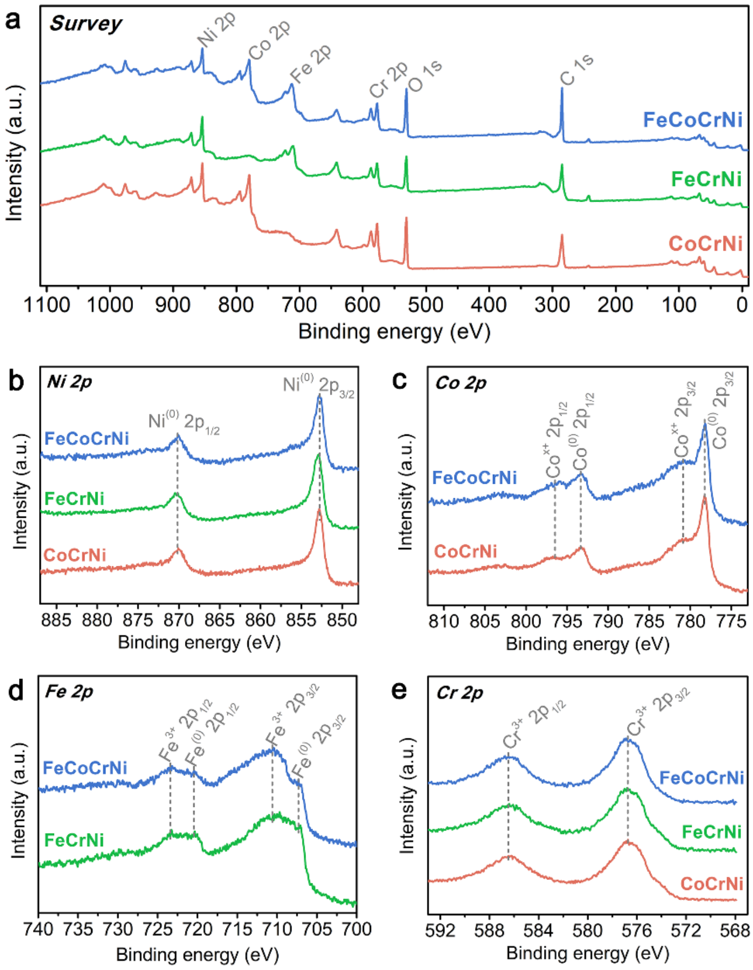

Supplementary figure 8 | XPS spectra of MCA films. (**a**) Survey spectra. (**b-e**) High resolution (**b**) Ni, (**c**) Co, (**d**) Fe, and (**e**) Cr 2*p* spectra. Details are discussed in Supplementary Note 1.

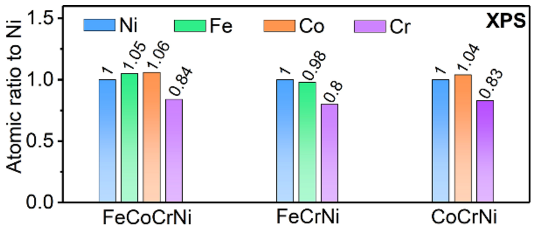

Supplementary figure 9 | Atomic ratios of the elements in MCA films based on XPS results. The ratios were normalized by Ni component.

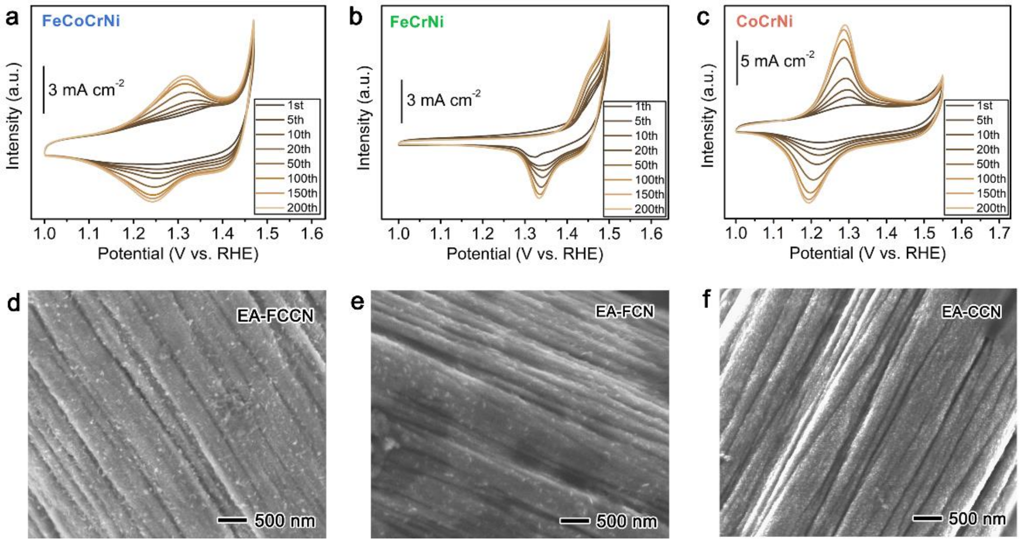

Supplementary figure 10 | Electrochemical activation of MCA films coated on CCs. (**a-c**) CV scanning curves and (**d-f**) corresponding SEM images of electrochemical activated MCA films.

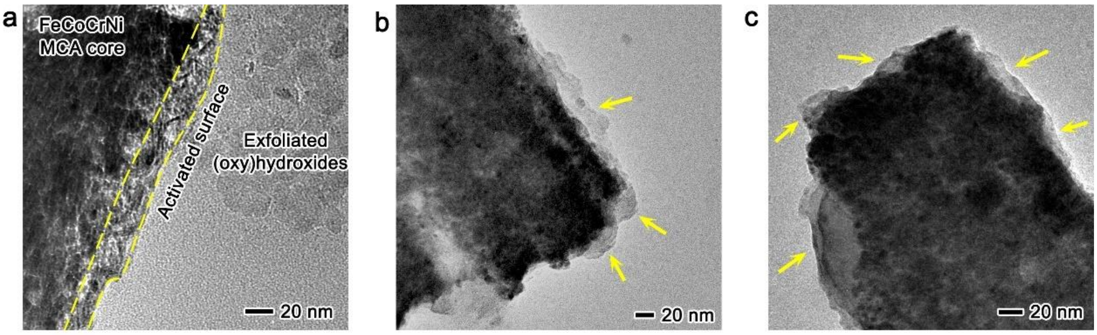

Supplementary figure 11 | TEM images of EA-FCCN catalyst.

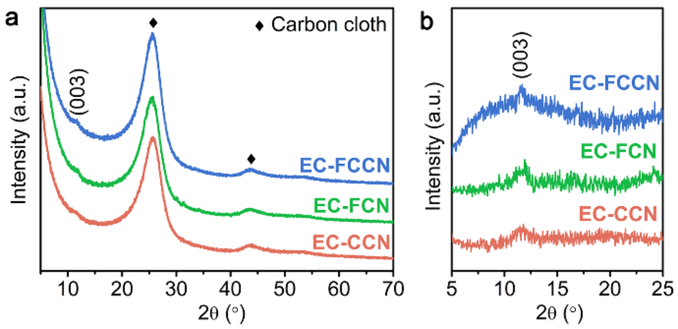

Supplementary figure 12 | XRD patterns of electrochemical activated MCA films. (**a**) Raw data. (**b**) Partially enlarged patterns with subtraction of CC background.

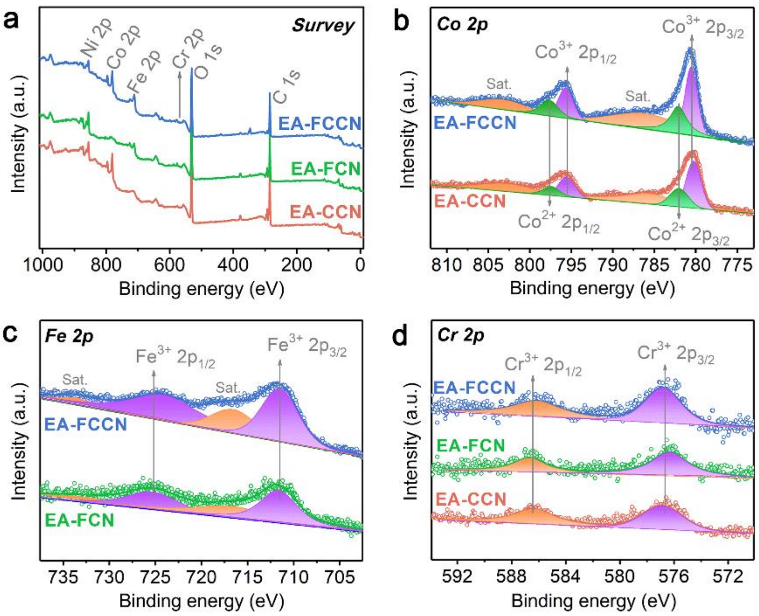

Supplementary figure 13 | XPS spectra of electrochemical activated MCA films. (**a**) Survey spectra. (**b-d**) High resolution (**b**) Co, (**c**) Fe, and (**d**) Cr 2*p* spectra. Corresponding Ni 2*p* and O 1*s* XPS spectra are shown in Fig. 1f and 1g, respectively. Details are discussed in Supplementary Note 2.

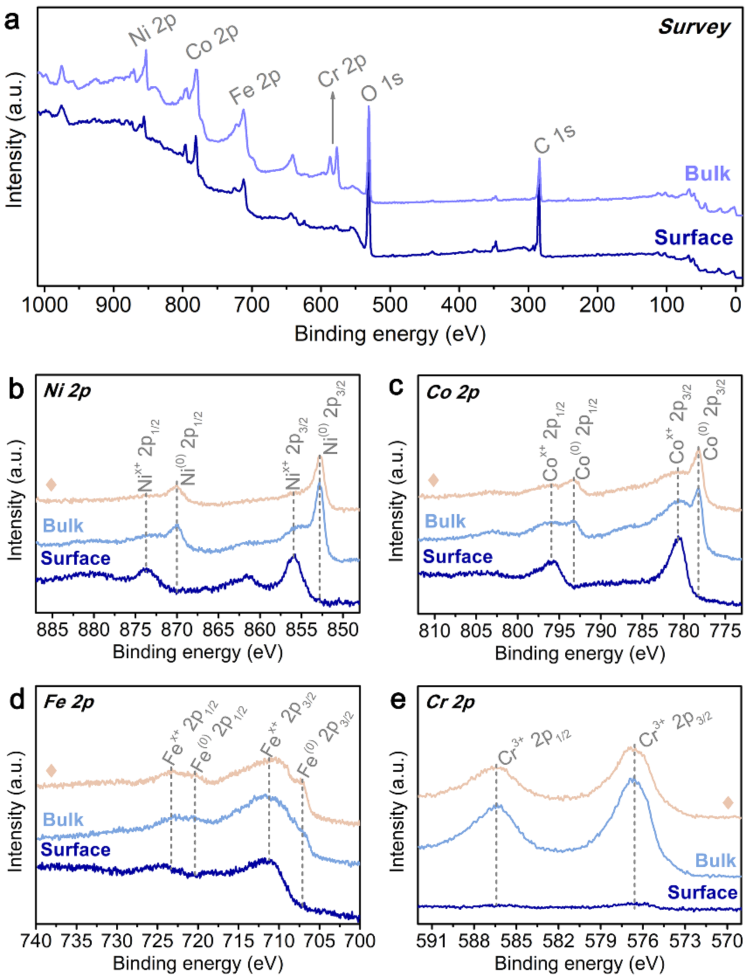

Supplementary figure 14 | XPS spectra of EA-FCCN film after Ar^{+} plasma etching for 10 min. (**a**) Survey spectra. (**b-d**) High resolution (**b**) Ni, (**c**) Co, (**d**) Fe, and (**e**) Cr 2*p* spectra. The light orange curves (labelled to ♦) represent the related metal 2*p* XPS spectra of pristine FeCoCrNi MCA film.

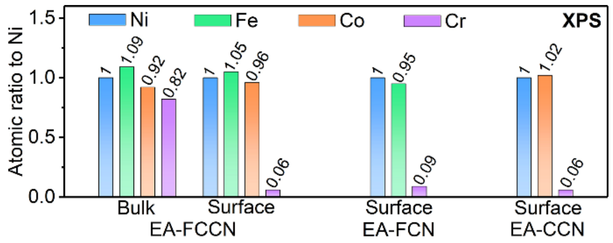

Supplementary figure 15 | Atomic ratios of the elements in electrochemical activated MCA films based on XPS results. The ratios were normalized by Ni component. The atomic ratios of bulk of EA-FCCN film were determined from XPS results through Ar^+ plasma etching for 10 min.

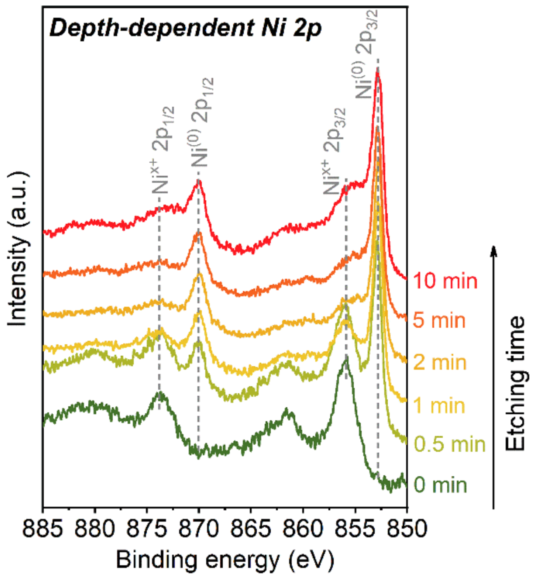

Supplementary figure 16 | Depth-dependent Ni 2*p* XPS spectra of EA-FCCN with different Ar^+ plasma etching times. The spectrums of 0 and 10 min are abstracted from Supplementary Fig. 14b (namely, surface and bulk spectrums, respectively).

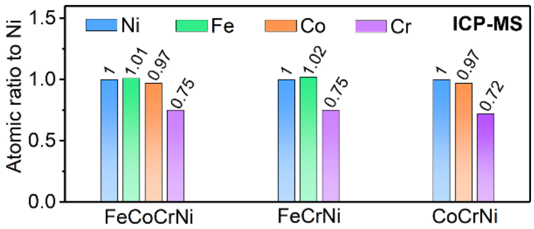

Supplementary figure 17 | Atomic ratios of the elements in electrochemical activated MCA films determined by ICP-MS results. The atomic ratios were normalized by Ni component.

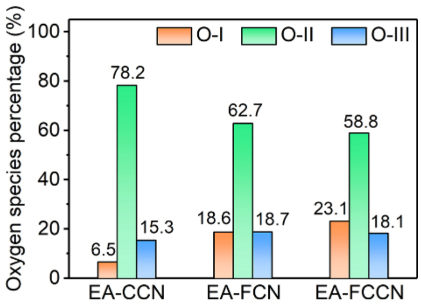

Supplementary figure 18 | Quantitative analysis of different oxygen species of electrochemical activated MCA films based on the fitting result of O 1*s* XPS spectra in Fig. 1g.

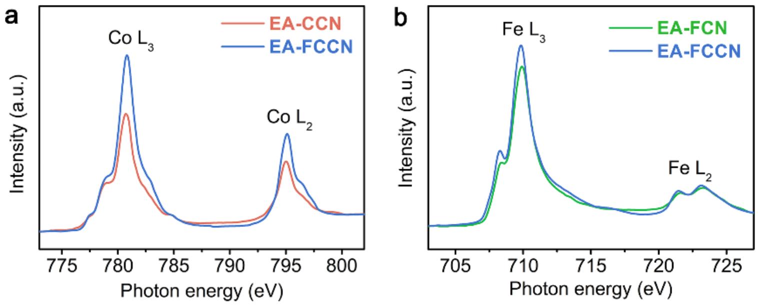

Supplementary figure 19 | Metal *L*-edge sXAS spectra of electrochemical activated MCA films. (**a**) Co *L*-edge. (**b**) Fe *L*-edge.

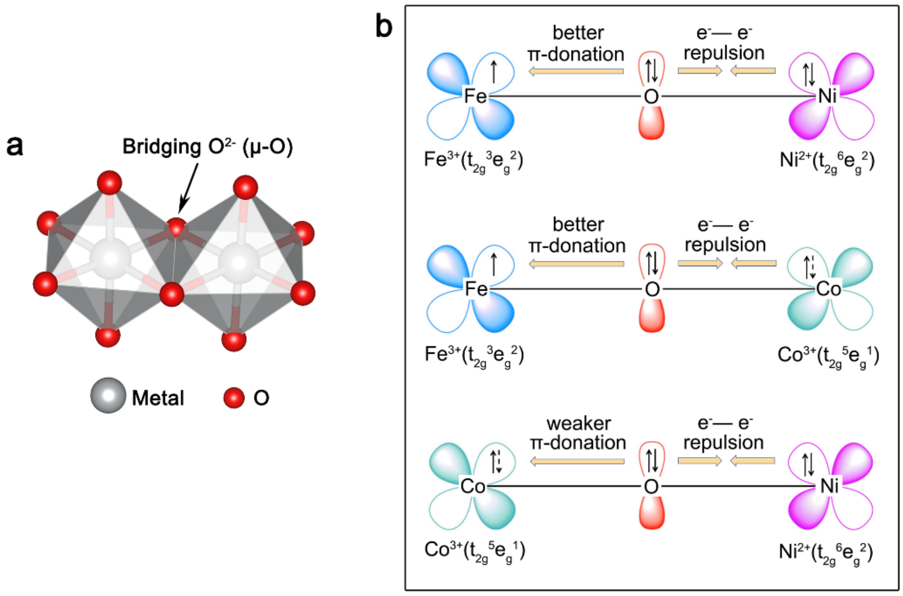

Supplementary figure 20 | Schemes of *μ*-bridged oxygen. (**a**) Schematic representation of two *μ*-bridged MO_6 octahedrons in layered metal (oxy)hydroxide structure. (**b**) Schematic representations of PET in Fe^{3+}-O-Ni^{2+}, Fe^{3+}-O-Co^{3+}, and Co^{3+}-O-Ni^{2+} moieties through bridging O (*μ*-O) atom, respectively, where left metal atom serves as the electron acceptor.

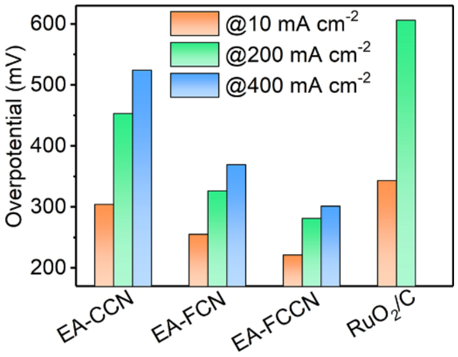

Supplementary figure 21 | Overpotentials to reach different current densities for various catalysts.

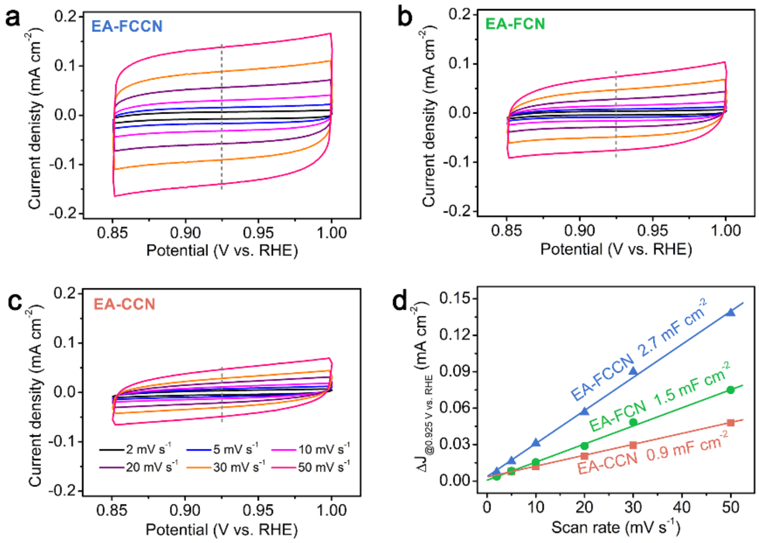

Supplementary figure 22 | The determination of C_{dl} for various catalysts. (**a-c**) CV scanning curves of (**a**) EA-FCCN, (**b**) EA-FCN, and (**c**) EA-CCN catalysts in O_2-saturated 1-M KOH solution at different scan rates in the non-Faradaic potential region (0.85-1.0 V *versus* RHE). (**d**) Capacitive current density differences ($\Delta J = (J_+ - J_-)/2$) at 0.925 V *versus* RHE as a function of scan rate. The linear slope is equivalent to C_{dl}.

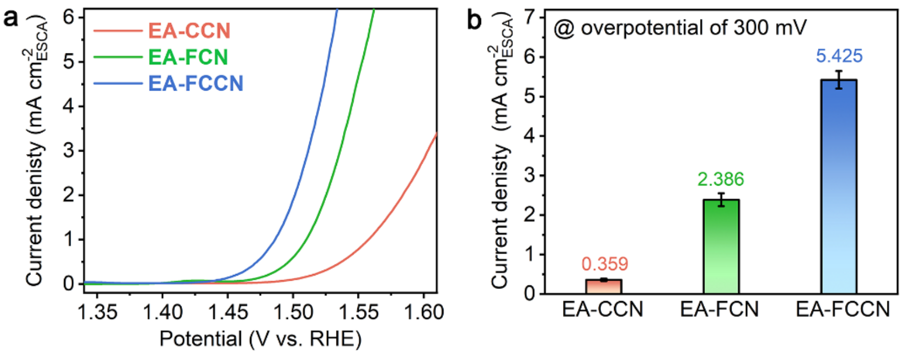

Supplementary figure 23 | Normalized specific activities by ECSAs of various catalysts. (**a**) The corresponding LSV polarization curves. (**b**) Current density values at an overpotential of 300 mV.

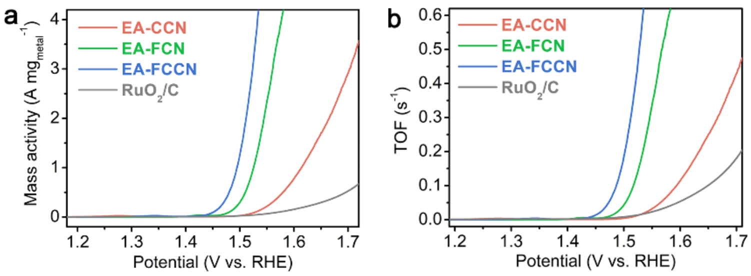

Supplementary figure 24 | Calculated LSV polarization curves of intrinsic activities for various catalysts. (**a**) Mass activities. (**b**) TOFs

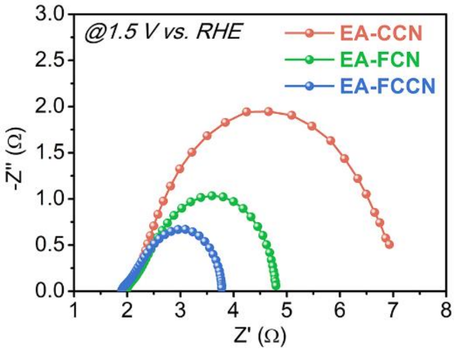

Supplementary figure 25 | Nyquist plots of various catalysts at 1.5 V *versus* RHE in O_2-saturated 1-M KOH solution.

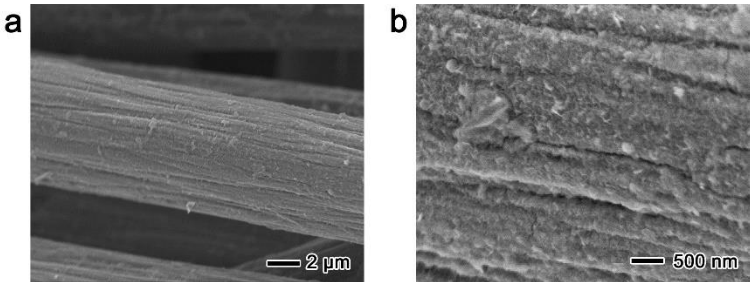

Supplementary figure 26 | SEM images with different magnification of EA-FCCN catalyst after chronoamperometric test for 20 h. (**a**) low magnification. (**b**) high magnification.

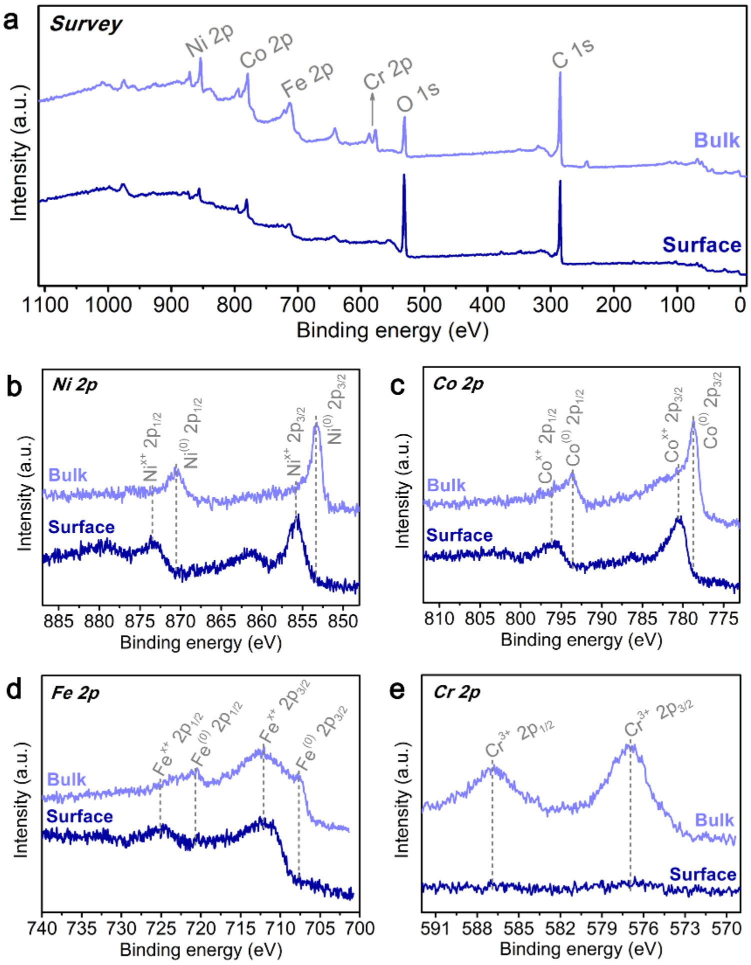

Supplementary figure 27 | XPS spectra of EA-FCCN catalyst after chronoamperometric test for 20 h. (**a**) Survey spectra. (**b-d**) High resolution (**b**) Ni, (**c**) Co, (**d**) Fe, and (**e**) Cr 2*p* spectra. Bulk spectra were collected after Ar^+ plasma etching for 10 min.

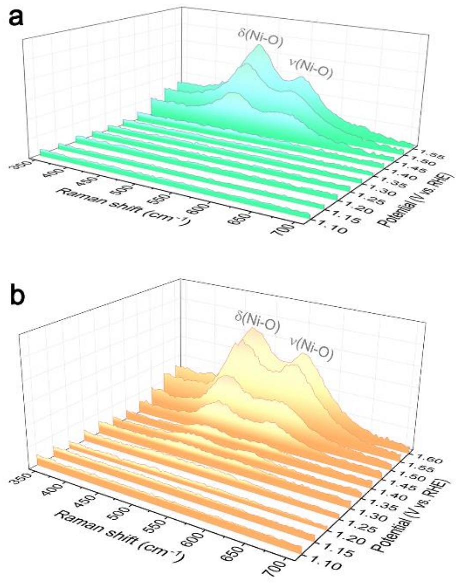

Supplementary figure 28 | Electrochemical *in situ* Raman spectra at the range of 350-700 cm^{-1} of various catalysts. (**a**) EA-FCN. (**b**) EA-CCN. Spectra were collected at the operated potentials of 1.1-1.55 V and 1.1-1.6 V *versus* RHE for EA-FCN and EA-CCN, respectively.

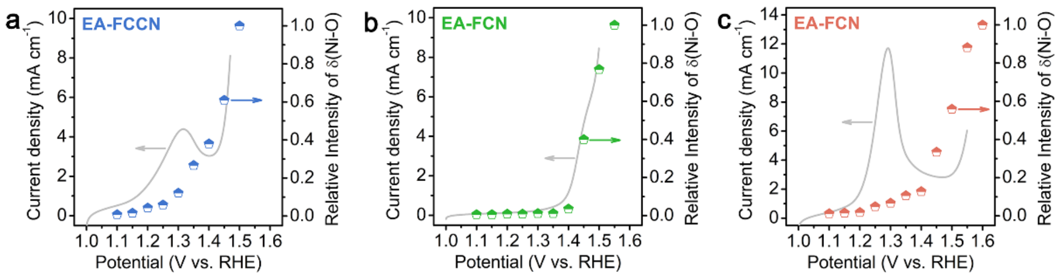

Supplementary figure 29 | Electrochemical *in situ* Raman signals of *δ*(Ni-O) together with the corresponding positive CV scanning portions. (**a**) EA-FCCN. (**b**) EA-FCN. (**c**) EA-CCN. The Raman signals were normalized by signal intensities at the highest operated potentials.

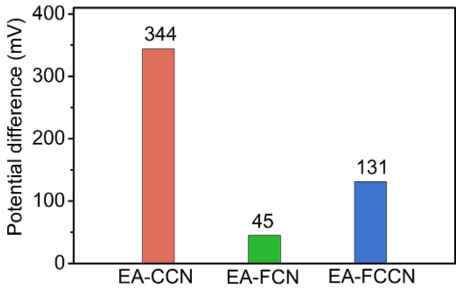

Supplementary figure 30 | The potential differences of various catalysts between Ni^{2+} oxidation peak and initiation of OER process (reaching a current density of 10 mA cm^{-1}).

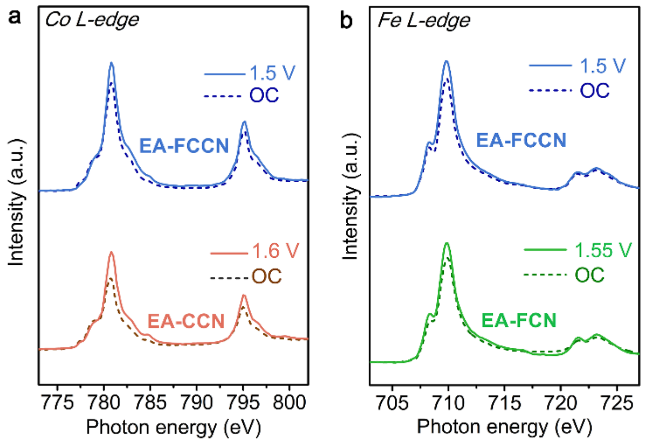

Supplementary figure 31 | *Ex situ* normalized metal *L*-edge sXAS spectra of various catalysts at different operated potentials. (**a**) Co *L*-edge. (**b**) Fe *L*-edge.

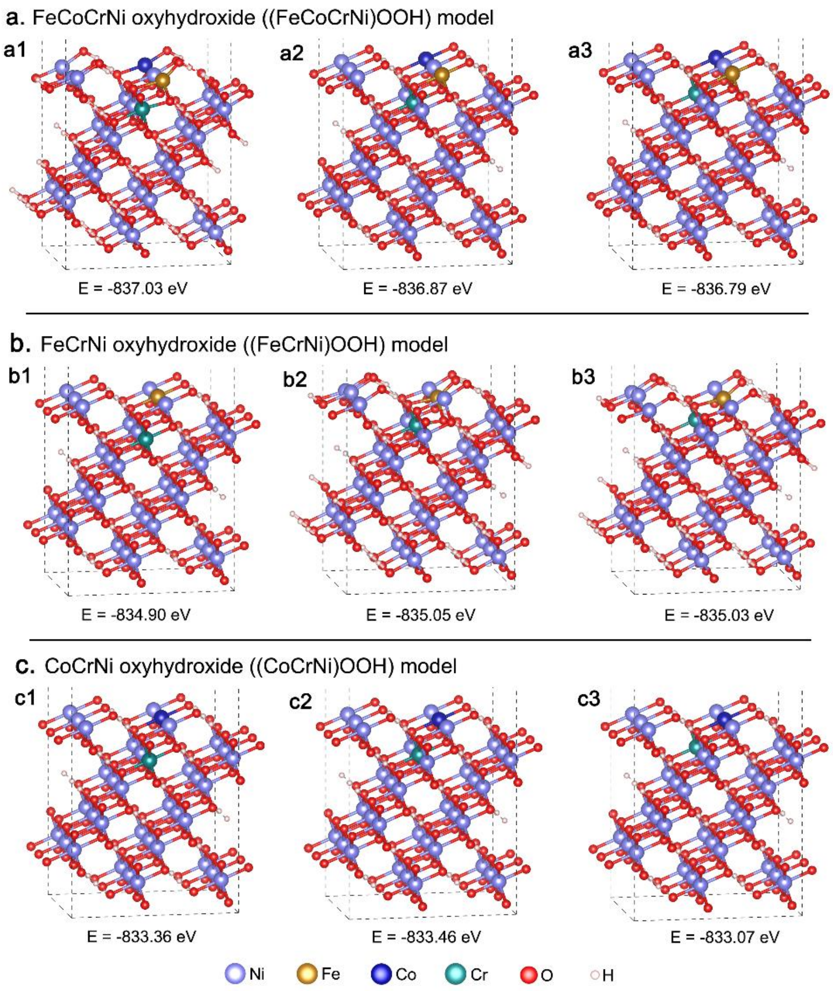

Supplementary figure 32 | Optimized configurations with their enthalpies for DFT simulation. (**a**) (FeCoCrNi)OOH model. (**b**) (FeCrNi)OOH model. (**c**) (CoCrNi)OOH model.

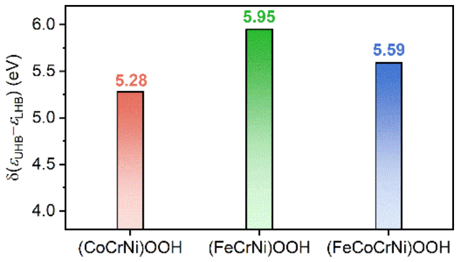

Supplementary figure 33 | The determined *U* of localized Fe/Co-neighboring Ni sites in different models. *U* is qualitatively determined by the energy difference between ε_{LHB} and ε_{UHB} (*i.e.*, $\delta(\varepsilon_{UHB} - \varepsilon_{LHB})$ in the figure).

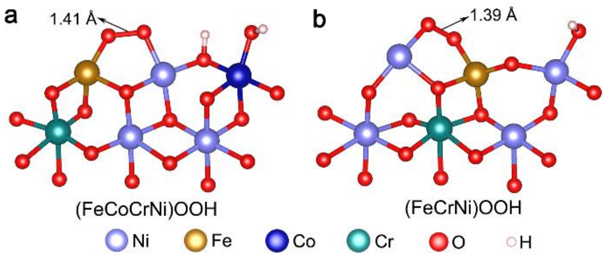

Supplementary figure 34 | The calculated O-O bond length of lattice oxygen coupled peroxo-like oxygen species in different models. (**a**) (FeCoCrNi)OOH model. (**b**) (FeCrNi)OOH model.

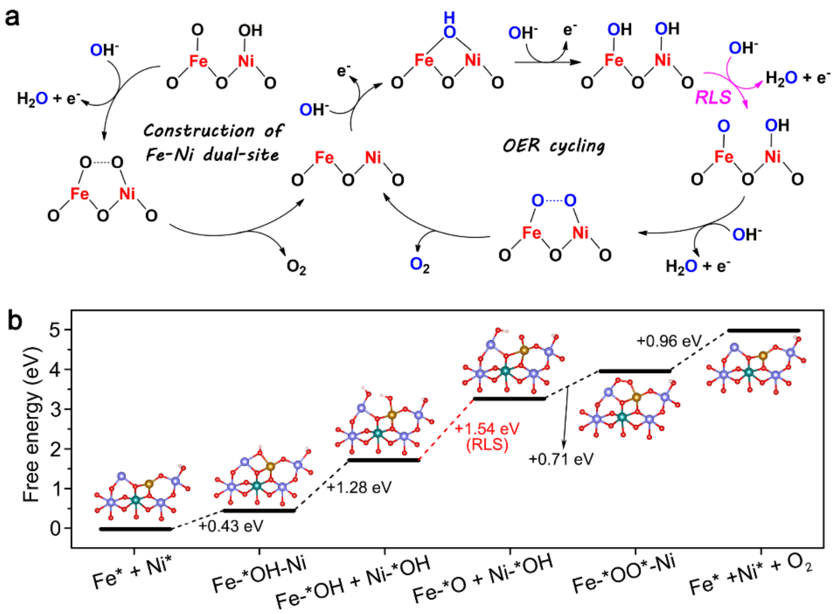

Supplementary figure 35 | OER cycling on (FeCrNi)OOH model. (**a**) Schematic illustration of the proposed overall OER pathway for (FeCrNi)OOH model, *i.e.*, EA-FCN catalyst. (**b**) The simulated free energy diagrams of OER cycling on (FeCrNi)OOH model with Fe-Ni dual-site as ultimate catalytic center.

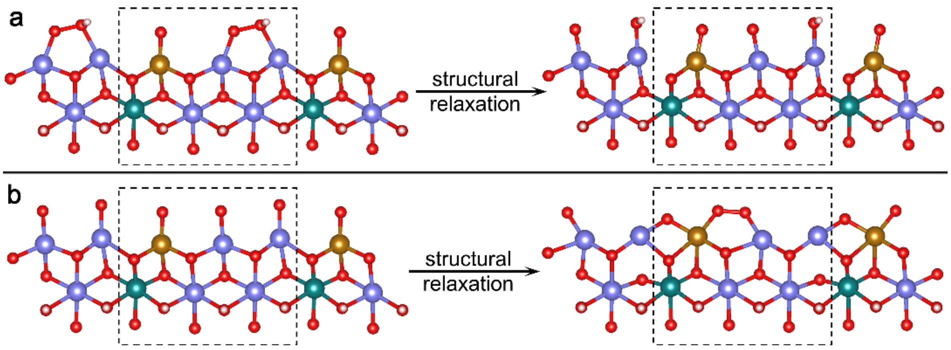

Supplementary figure 36 | Evaluating the possibility of Ni-Ni dual-site as catalytic center on (FeCrNi)OOH model. (**a**) Compulsive construction of O-O bond at Ni-Ni dual-site and its evolution after structural relaxation. (**b**) Deleting all surface hydrogen atoms and its evolution after structural relaxation.

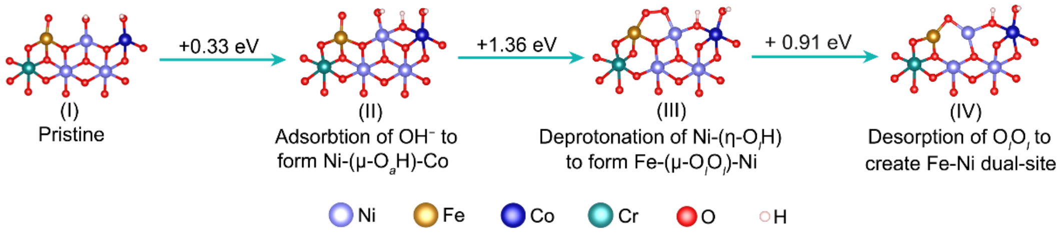

Supplementary figure 37 | The configuration illustrations of initial evolution of surface oxygen species for constructing Fe-Ni dual-site on (FeCoCrNi)OOH model.

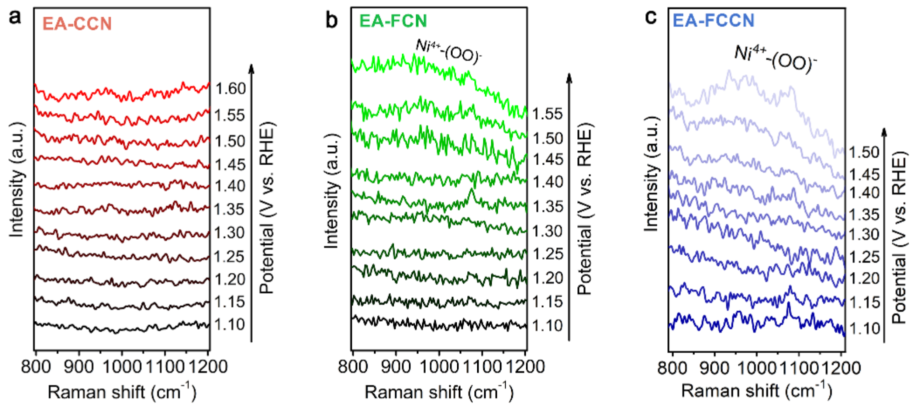

Supplementary figure 38 | Electrochemical *in situ* Raman spectra at the range from 800-1200 cm^{-1} of various catalysts. (**a**) EA-CCN. (**b**) EA-FCN. (**c**) EA-FCCN. Spectra were collected at the operated potentials of 1.1-1.6, 1.1-1.55 and 1.1-1.5 V *versus* RHE for EA-CCN, EA-FCN and FA-FCCN catalysts, respectively.

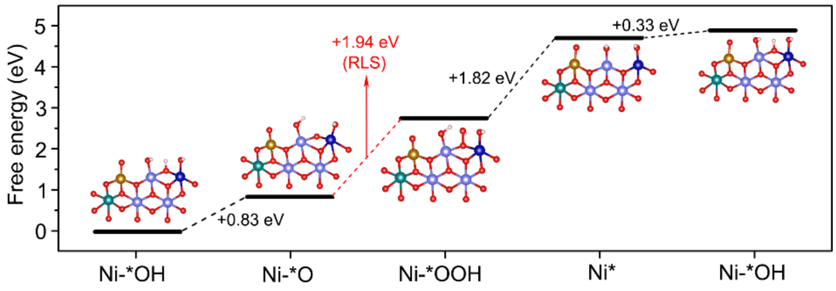

Supplementary figure 39 | The simulated energy barrier diagram of conventional AEM pathway on (FeCoCrNi)OOH model.

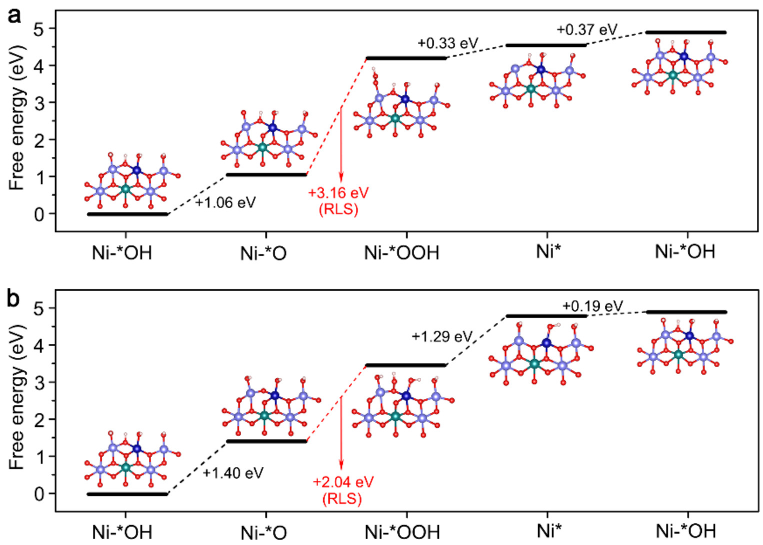

Supplementary figure 40 | The simulated energy barrier diagrams of OER cycling of different pathways on (CoCrNi)OOH model. (**a**) LOM pathway through Ni-η-O_lH deprotonation. (**b**) AEM pathway through μ-O_aH deprotonation.

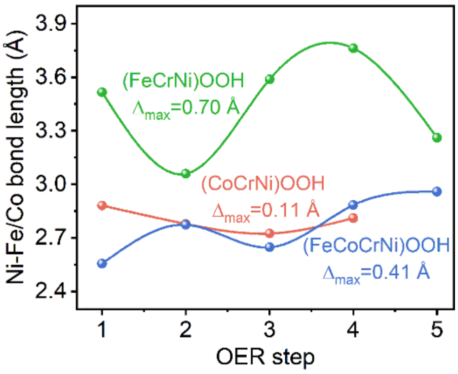

Supplementary figure 41 | The variation of metal-metal (Ni-Fe/Co) bond length during proposed OER cycling of various models. (LOM pathway for (FeCoCrNi)OOH and (FeCrNi)OOH models, and AEM pathway for (CoCrNi)OOH model).

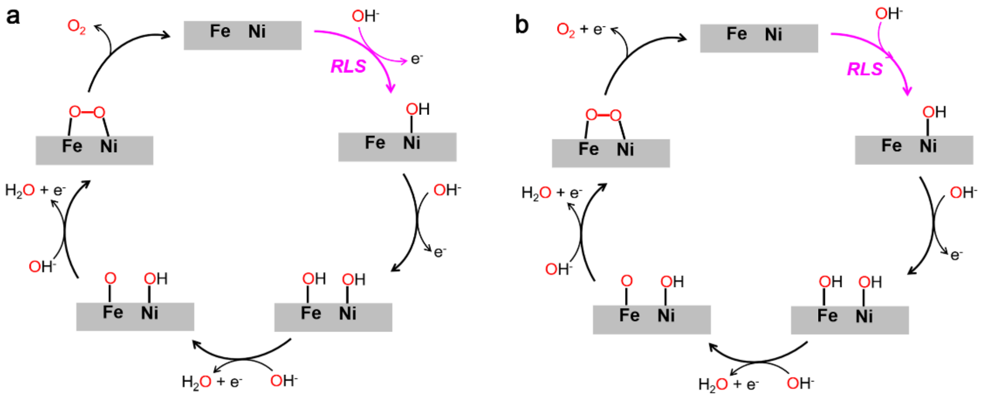

Supplementary figure 42 | Schematic representations of OER cycling on (FeCoCrNi)OOH model. (**a**) Concerted electron-proton transfer step. (**b**) Non-concerted electron-proton transfer step.

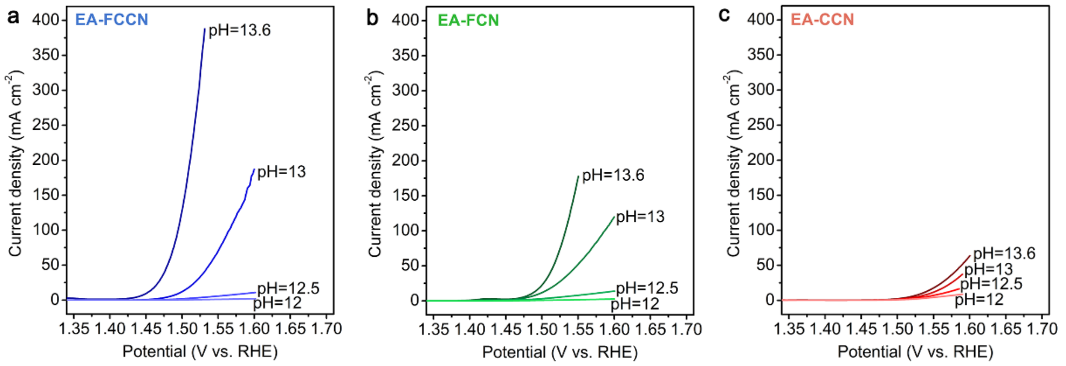

Supplementary figure 43 | pH dependence LSV polarization curves of various catalysts. (**a**) EA-FCCN. (**b**) EA-FCN. (**c**) EA-CCN.

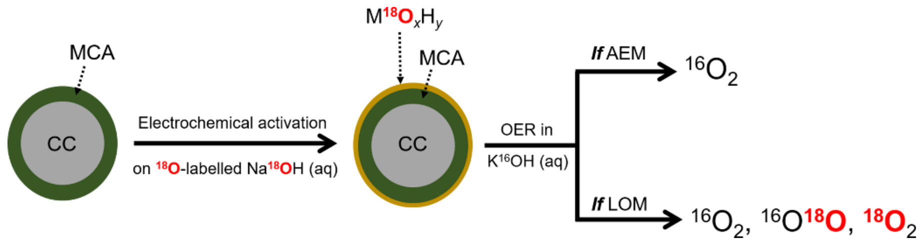

Supplementary figure 44 | Schematic illustration of our designed ^{18}O isotope-labelled experiment.

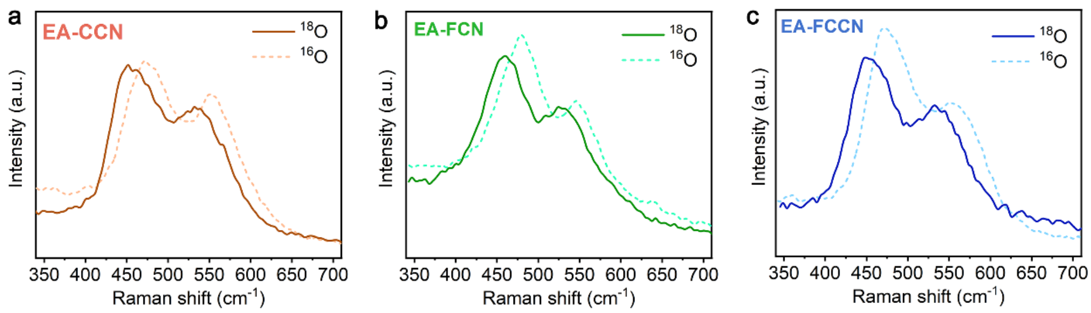

Supplementary figure 45 | Electrochemical *in-situ* Raman spectra of ^{18}O-labelled catalysts measured at the operated potentials. (**a**) EA-CCN at 1.6 V, (**b**) EA-FCN at 1.55 V, and (**c**) EA-FCCN at 1.5 V *versus* RHE, respectively. The Ni-O signals of NiOOH structure exhibit the red-shift of 20-25 cm^{-1}, meaning that the dynamically reconstructed metal oxyhydroxides are successfully ^{18}O-labelled (*i.e.*, $M^{18}O_xH_y$ is formed)[1].

Supplementary Tables.

Supplementary Table 1 | ICP-MS results of various samples.[a]

Sample	Elemental concentration (μg mL^{-1})			
	Ni	Fe	Co	Cr
FeCoCrNi	1.964	1.888	1.856	1.410
FeCrNi	2.035	1.859	\	1.406
CoCrNi	2.225	\	2.079	1.537
EA-FCCN	1.883	1.809	1.836	1.252
EA-FCN	1.908	1.851	\	1.267
EA-CCN	2.087	\	2.035	1.331

[a]A piece of MCA coated CC with area of 1 cm^2 was firstly immersed into 3-mL aqua regia solution for two days to ensure the total dissolution of metal components. The as-prepared solution was further diluted to 15 mL before ICP-MS detection. Three parallel CCs were synchronously measured for each sample to minimize the error.

Supplementary Table 2 | Summary of assignments of XPS peaks in various MCA films[1].

Element	Metal state peak (eV)		Oxidation state peak (eV)	
	$2p_{3/2}$	$2p_{1/2}$	$2p_{3/2}$	$2p_{1/2}$
Ni	852.7	870.2	\	\
Co	778.2	793.2	780.8	796.4
Fe	707.3	720.5	710.6	723.3
Cr	\	\	576.7	586.4

Supplementary Table 3 | The standard reduction potentials ($E°$) *versus* standard hydrogen electrode (SHE) of various metals[3].

Reduction half reaction	$E°$ (V *versus* SHE)
$Ni^{2+} + 2e^{-} \rightarrow Ni(s)$	−0.25
$Co^{2+} + 2e^{-} \rightarrow Co(s)$	−0.28
$Fe^{2+} + 2e^{-} \rightarrow Fe(s)$	−0.44
$Cr^{3+} + 2e^{-} \rightarrow Cr(s)$	−0.74

Supplementary Table 4 | Summary of assignments of metal 2p XPS peaks in various electrochemical activated MCA films[2].

Element	M^{2+} peak (eV)		M^{3+} peak (eV)	
	$2p_{3/2}$	$2p_{1/2}$	$2p_{3/2}$	$2p_{1/2}$
Ni	855.5	872.9	857.0	875.4
Co	782.1	797.6	780.5	795.5
Fe	\	\	711.6	725.2
Cr	\	\	576.7	586.4

Supplementary Table 5 | The determined C_{dl}s and ECSAs of various catalysts.

Catalyst	C_{dl} (mF cm^{-2})	ECSA (cm^{-2})[a]
EA-CCN	0.9	22.5
EA-FCN	1.5	37.5
EA-FCCN	2.7	67.5

[a] ECSAs are determined by the equation of ECSA = $C_{dl}/C_s \times S$, where C_s is the capacitance of an atomically smooth planar surface (0.04 mF cm^{-2} in alkaline media)[4], and S is the electrode area.

Supplementary Table 6 | Comparison of alkaline OER performance with previously reported earth-abundant catalysts.

Catalyst[a]	η (mV) @10 mA cm^{-2}	Tafel slope (mV dec^{-1})	OER activity @η = 300 mV		Reference
			Mass activity (A g^{-1})	TOF (s^{-1})	
**EA-FCCN/CC**	**221**	**38.7**	**3601**	**0.483**	**This work**
EA-FCN/CC	255	42.6	1183	0.150	This work
EA-CCN/CC	304	51.4	101	0.014	This work
Exfoliated 2D FeNi-LDH/GCE	302	40	/	0.05	5
$Ni_{0.75}V_{0.25}$-LDH/GCE	318	50	~400[c]	0.054[c]	6
2D-NiCo-MOF/CF	189	42	36	~0.3	7
G-FeCoW/GCE	223	37	1175	0.46	8
FeNiCr-LDH/Cu nanorrays	200	28	250[d]	0.04[d]	9
$NiCeO_xH_y$/NF	177	22	406.8[e]	/	10
FeNiV (oxy)hydroxide/CFP	200	39	/	0.574	11
Lattice-strained NiFe-MOFs/NF	210[b]	68	2000	/	12
CoFeW nanoclusters/CC	192	36	/	0.377[d]	13
CuNiFe hydr(oxy)oxide/NF	181	44	1464.5	/	14
NiO-NiFe LDH/CFP	215	32	305	0.32	15
NiTe/NiS/NF	209	49	/	0.178	16

[a] CC = carbon cloth; CFP = carbon fiber paper; NF = Ni foam; CF = Cu foam; GCE = glassy carbon electrode.

[b] Current density = 200 mA cm^{-2}.

[c] η = 350 mV. [d] η = 250 mV. [e] η = 270 mV.

Supplementary Notes.

Supplementary Note 1 | More discussions for XPS results of pristine MCA films.

As depicted in XPS spectra of MCA films (Supplementary Fig. 8), all of the MCA films exhibit the metallic feature together with inevitable surface oxidation. The peak assignments of high resolution metal 2*p* XPS spectra are summarized in Supplementary Table 2[2]. The oxidized degree of various metal elements can be well distinguished, which positively correlates to the related metal reduction potentials (Supplementary Table 3)[3]. For Ni component with the highest reduction potential of Ni^{2+}/Ni (−0.25 V *versus* SHE), two spin-orbit peaks at binding energies of 852.7 and 870.2 eV are assigned to $2p_{3/2}$ and $2p_{1/2}$ of metallic Ni species, respectively, as seen in Supplementary Fig. 8b. For Co component (reduction potential of Co^{2+}/Co is −0.28 V *versus* SHE), even though metallic Co species still prominently exists, the oxidation state species (binding energies of 780.8 and 796.4 eV) has been clearly discerned from Co 2*p* XPS spectra (Supplementary Fig. 8c). Fe component with a further lower reduction potential (*i.e.*, Fe^{2+}/Fe, −0.44 V *versus* SHE) gives rise to more serious oxidation. The main peaks located at binding energies of 710.6 and 723.3 eV are indexed to oxidized Fe species (Fe^{x+}, $x = 2, 3$), while the subordinate shoulder peaks at lower binding energies of 707.3 and 720.5 eV are assigned to be metallic Fe species (Supplementary Fig. 8d). In the case of Cr component, the lowest reduction potential of Cr^{3+}/Cr (−0.74 V *versus* SHE) induces that Cr is almost fully oxidized to Cr^{3+} species, where the related $2p_{3/2}$ and $2p_{1/2}$ peaks are located at binding energies of 576.7 and 586.4 eV, respectively (Supplementary Fig. 8e). Moreover, quantitative elemental analysis results based on XPS spectra (Supplementary Fig. 9) also uncover the nominally equiatomic feature in all of the MCA films with slightly lower Cr concentration, which agrees with SEM-EDS and ICP-MS results (Supplementary Fig. 7).

Supplementary Note 2 | More discussions for XPS results of electrochemical activated MCA films.

A pre-activation process was implemented for as-obtained MCA films using electrochemical CV scanning procedure. The electrochemical activated MCA films were also investigated by XPS spectra (see Fig. 1f and Supplementary Fig. 13). Their characteristic peaks are largely differentiated with pristine MCA films, where all of the metal components exhibit the thorough oxidation. The related deconvolution results are summarized in Supplementary Table 4[2]. To be specific, Ni 2*p* XPS spectra exhibits the main peak of Ni^{2+} species in (oxy)hydroxide at the binding energies of 855.5 and 872.9 eV. The peaks at 857.0 and 875.4 eV indicate the secondarily existed Ni^{3+} species. For Co component, the case is inverse, where Co^{3+} species is dominantly emerged (binding energies of 780.5 and 795.5 eV), along with subordinate Co^{2+} species (binding energies of 782.1 and 797.6 eV). In the case of Fe and Cr components, we discern that they are fully oxidized to M^{3+} species. We also observe that Cr component is largely leached (less than 10% retaining) due to its obviously decreased intensity and quantitative result (Supplementary Fig. 15). Moreover, this electrochemical activation can be considered as a surface engineering without altering the bulk matrix, as the bulk XPS spectrums (detected after Ar^{+} plasma etching, see Supplementary Fig. 14 and 16) still show the metallic feature, similar to those of pristine MCA film.

Supplementary Note 3 | Additional statements for partial electron transfer (PET) in M-O-M moiety (M = Fe, Co, and Ni).

As illustrated in Supplementary Fig. 20, PET can be triggered in different metal center with bridging O^{2-} (μ-O) through π-donation[7,11,17]. Specifically, three M-O-M moieties (*i.e.*, Fe^{3+}-O-Ni^{2+}, Co^{3+}-O-Ni^{2+}, and Fe^{3+}-O-Co^{3+}) are modeled. Given the O_h symmetry of hexa-coordinated MO_6, the valence electronic configurations of Ni^{2+}, Co^{3+}, and Fe^{3+} species can be typically determined to be $3d^8$ ($t_{2g}^6e_g^2$), $3d^6$ (intermediate-spin (IS), $t_{2g}^5e_g^1$)[18], and $3d^5$ (high-spin (HS), $t_{2g}^3e_g^2$)[19], respectively. Considering their different electronic configuration of 3*d*-orbitals (especially for electronic occupancy in π-symmetric t_{2g}-orbitals), the strength of PET can be distinguished, scilicet, Fe^{3+}-O-Ni^{2+} > Fe^{3+}-O-Co^{3+} > Co^{3+}-O-Ni^{2+} (left metal atom serves as the electron acceptor). Fe^{3+}-O-Ni^{2+} moiety exhibits the strongest PET because the fully occupied π-symmetric *d*-orbitals of Ni^{2+} (*i.e.*, t_{2g}^6) trigger the strongest e^--e^- repulsion with *p*-orbitals of μ-O, forcing the PET from Ni^{2+} to Fe^{3+} (Supplementary Fig. 20b, top). Similarly, PET can still be efficiently triggered in Fe^{3+}-O-Co^{3+} moiety but is slightly weaker due to t_{2g}^5 occupancy for Co^{3+} (Supplementary Fig. 20b, middle). In contrast, as both Ni^{2+} and Co^{3+} act e^--e^- repulsion with μ-O, the PET is the weakest in Co^{3+}-O-Ni^{2+} moiety (Supplementary Fig. 20b, bottom).

In terms of our samples with multimetal components, the PET can be deciphered by the combination of different moieties. As the Fe, Co, and Ni components are still nominally equiatomic and homogeneiously dispersed in electrochemical activated MO_xH_y, the arrangement of metal atoms can be simplified to be $\left(M_1\text{-O-}M_2\right)_n$ or $\left(M_1\text{-O-}M_2\text{-O-}M_3\right)_n$. As a result, the PET in our electrochemical activated MCA films (*i.e.*, EA-FCCN, EA-FCN, and EA-CCN) and the relative electron density can be summarized in the following:

For Ni component, EA-FCCN contains both Fe^{3+}-O-Ni^{2+} and Co^{3+}-O-Ni^{2+} moieties; while EA-FCN and EA-CCN only contain Fe^{3+}-O-Ni^{2+} and Co^{3+}-O-Ni^{2+} moieties,

respectively. As a result, the electron density tendency in Ni should be EA-CCN > EA-FCCN > EA-FCN, videlicet, the white line intensity tendency of Ni *L*-edge sXAS spectra should be EA-FCN > EA-FCCN > EA-CCN.

For Co component, EA-FCCN contains Fe^{3+}-O-Co^{3+} and Co^{3+}-O-Ni^{2+} moieties; and EA-CCN contains Co^{3+}-O-Ni^{2+} moiety. As a result, the electron density tendency in Co should be EA-CCN > EA-FCCN, videlicet, the white line intensity tendency of Co *L*-edge sXAS spectra should be EA-FCCN > EA-CCN.

For Fe component, EA-FCCN contains Fe^{3+}-O-Ni^{2+} and Fe^{3+}-O-Co^{3+} moieties; and EA-FCN contains Fe^{3+}-O-Ni^{2+} moiety. As a result, the electron density tendency in Fe is EA-FCN > EA-FCCN, videlicet, the white line intensity tendency of Fe *L*-edge sXAS spectra is EA-FCCN > EA-FCN.

The determination of electron density fluctuation based on PET effect well matches with experimental sXAS fingerprints (Fig. 1h and Supplementary Fig. 19). Notably that the elucidation of electron density tendency based on PET is qualitative because those models are idealized, and the physical truth is much more complicated (considering multimetal composition and changeable electronic configurations). However, we emphasize that those qualitative results are reasonable and sufficient to support our arguments.

Finally, we also hold that the PET effect can be still effective in highly oxidized states of metal species, as their different electronic configuration of 3*d*-orbitals. To be specific, Ni^{3+}, Ni^{4+}, Co^{4+}, and Fe^{4+} species are typically considered to exhibit the electronic configurations of $3d^7$ (low-spin (LS), $t_{2g}^6e_g^1$)[20], $3d^6$ (LS, $t_{2g}^6e_g^0$)[20], $3d^5$ (IS, $t_{2g}^4e_g^1$)[21], and $3d^4$ (HS, $t_{2g}^3e_g^1$)[22], respectively. The valence variations typically alter the occupancy of e_g-orbitals rather than π-symmetric t_{2g}-orbitals. Hence, electrons can also transfer from $Ni^{3/4+}$ species to Co and/or Fe species through the proposed PET effect.

Supplementary Note 4 | Additional statements for the models of DFT simulations.

NiOOH structure exposed (10−14) lattice plane was chosen as the pristine host matrix to establish the catalyst models and perform the DFT simulations. The choice of oxyhydroxide matrix is reasonable as it has been verified as the ultimate structure to offer the veritable active sites[8,23-25]. Besides, it has been reported that (10−14) terminated surface is more stable and active when exposed to serve as catalytic surface[23,24]. To simulate the local structure of our catalysts, different metal atoms were alternatively doped on the (sub)surface to substitute for Ni atoms, as shown in Supplementary Fig. 32. Cr atom was set into subsurface and did not participate in OER process as its leaching based on our experimental results. We choose the optimized models with the lowest enthalpies to serve as our candidates for further simulation of OER pathway, as shown in Supplementary Fig. 32 (namely, a1 configuration for (FeCoCrNi)OOH model, b2 configuration for (FeCrNi)OOH model, and c2 configuration for (CoCrNi)OOH model, respectively). It should be noted that H in surface Fe-OH is desorbed and connected with interlayered Ni-O after configuration optimization with energy convergence, while surface Ni-OH and Co-OH can be well maintained. As such, the surface structure is set to contain Fe-O, Ni-OH and/or Co-OH for initial configurations. In the case of simulation of OER pathway on various models, different configurations with adsorbing oxygen species or deprotonating were tested until energy convergence to determine the preferential pathway. Notably that this is just a simplified approach as the simulations of real multimetal structure are largely hindered by their complicacy at the present stage[8]. Our simulations mainly focus on investigating the evolution local atomic structure where OER process occurs and the effect of different metal atoms on reaction pathway. We hold the simulated results can guide us to decipher the experimental evidences with in-depth understanding.

Supplementary Note 5 | Computational details of the energy barrier of OER cycling on different models.

To simulate the OER pathway and determine the reaction energy barrier, the computational hydrogen electrode (CHE) model described by Nørskov *et al.* was applied[26-28]. In this CHE model, the chemical potential of the proton-electron pair (*i.e.*, the free energy per H) is determined simply by the chemical potential of gas-phase H_2 as Supplementary Equation (1), which can be adjusted as a function of the applied potential:

$$\mu(H^+) + \mu(e^-) = 1/2\mu_{H2(g)} - eU \quad (1)$$

We used the chemical potential of vapor water at P = 0.035 bars and T = 300 K to describe H_2O molecule ($\mu_{H2O(l)} = \mu_{H2O(g)}$ | P = 0.035 bars, T = 300 K), as it is equilibrium with liquid water[8].

For (FeCoCrNi)OOH and (FeCrNi)OOH models, Fe-Ni dual-site after lattice oxygen oxidation was proposed as the catalytic center. We started the O_2-desorbed model (*i.e.*, Fe* + Ni*) to furnish the OER cycling through the following five elementary steps (Steps 1-5), including four electrochemical steps and one non-electrochemical O_2 desorption step[8,29]:

$$Fe^* + Ni^* + OH^- \rightarrow Fe^* + Ni^*\text{-}OH + e^- \text{ (Step 1)}$$

$$Fe^* + Ni^*\text{-}OH + OH^- \rightarrow Fe^*\text{-}OH + Ni^*\text{-}OH + e^- \text{ (Step 2)}$$

$$Fe^*\text{-}OH + Ni^*\text{-}OH + OH^- \rightarrow Fe^*\text{-}O + Ni^*\text{-}OH + H_2O(l) + e^- \text{ (Step 3)}$$

$$Fe^*\text{-}O + Ni^*\text{-}OH + OH^- \rightarrow Fe^*\text{-}OO\text{-}^*Ni + H_2O(l) + e^- \text{ (Step 4)}$$

$$Fe^*\text{-}OO\text{-}^*Ni \rightarrow Fe^* + Ni^* + O_2(g) \text{ (Step 5)}$$

As such, the Gibbs free energy changes (ΔG) for each step can be calculated as Supplementary Equations (2-6):

$$\Delta G_1 = \Delta G_{(Fe^* + Ni^*\text{-}OH)} - eU + \Delta G_{pH} \quad (2)$$

$$\Delta G_2 = \Delta G_{(Fe^*\text{-}OH + Ni^*\text{-}OH)} - \Delta G_{(Fe^* + Ni^*\text{-}OH)} - eU + \Delta G_{pH} \quad (3)$$

$$\Delta G_3 = \Delta G_{(Fe^*\text{-}O + Ni^*\text{-}OH)} - \Delta G_{(Fe^*\text{-}OH + Ni^*\text{-}OH)} - eU + \Delta G_{pH} \quad (4)$$

$$\Delta G_4 = \Delta G_{(Fe^*\text{-}OO\text{-}^*Ni)} - \Delta G_{(Fe^*\text{-}O + Ni^*\text{-}OH)} - eU + \Delta G_{pH} \quad (5)$$

$$\Delta G_5 = 4.92 - \Delta G_{(Fe^*\text{-}OO\text{-}^*Ni)} \quad (6)$$

where U is the potential measured against the normal hydrogen electrode (NHE) under standard conditions (T = 298.15 K, P = 1 bar, pH = 0). ΔG_{pH} is the correction of the free energy change in non-zero pH conditions based on the Nernst equation (*i.e.*, $\Delta G_{pH} = -k_B T\ln(10) \times \text{pH}$, where k_B is Boltzmann constant). The overall pathway was fixed by an energy barrier of 4.92 eV derived from experimental splitting energy of water molecule (namely, $H_2O(l) \rightarrow H_2(g) + 1/2O_2(g)$, ΔG_{H2O}^{exp} = 2.46 eV), as O_2 molecule can hardly be accurately described with DFT due to its complicated electronic states[27]. The Gibbs free energy differences of various intermediates were determined as Supplementary Equation (7), which includes zero-point energy (ΔZPE) and entropy corrections ($T\Delta S$):

$$\Delta G = \Delta E + \Delta ZPE - T\Delta S \quad (7)$$

where the energy differences (ΔE) can be calculated relative to the chemical potentials of $H_2O(l)$ and $H_2(g)$ by Supplementary Equations (8-11):

$$\Delta E_{(Fe^* + Ni^*\text{-}OH)} = E_{(Fe^* + Ni^*\text{-}OH)} - E_{(Fe^* + Ni^*)} - (\mu_{H2O(l)} - 1/2\mu_{H2(g)}) \quad (8)$$

$$\Delta E_{(Fe^*\text{-}OH + Ni^*\text{-}OH)} = E_{(Fe^*\text{-}OH + Ni^*\text{-}OH)} - E_{(Fe^* + Ni^*)} - (2\mu_{H2O(l)} - \mu_{H2(g)}) \quad (9)$$

$$\Delta E_{(Fe^*\text{-}O + Ni^*\text{-}OH)} = E_{(Fe^*\text{-}O + Ni^*\text{-}OH)} - E_{(Fe^* + Ni^*)} - (2\mu_{H2O(l)} - 3/2\mu_{H2(g)}) \quad (10)$$

$$\Delta E_{(Fe^*\text{-}OO\text{-}^*Ni)} = E_{(Fe^*\text{-}OO\text{-}^*Ni)} - E_{(Fe^* + Ni^*)} - (2\mu_{H2O(l)} - 2\mu_{H2(g)}) \quad (11)$$

It should be noted that we simulated the electrochemical steps with conventional concerted proton-electron transfer steps, despite that non-concerted proton-electron transfer process is more common for LOM pathway (see discussion in Supplementary Fig. 42)[29,30]. If non-concerted proton-electron transfer issue were considered, the O_2 desorption step (Step 5) would be Fe*-OO-*Ni → Fe* + Ni* + $O_2(g) + e^-$ and the RLS would not involve the electron transfer, as described in Supplementary Fig. 42b. Therefore, the OER activity is pH dependent when non-concerped proton-electron transfer process occurs. Unfortunately, currently developed DFT calculation methods cannot well simulate and distinguish their difference. Considering this, we directly adopted the Gibbs free energy barriers of RLS to compare priority of different pathways

rather than the theoretical overpotential. It should be point out that this treatment does not influence our arguments towards preferential OER pathway.

In terms of (CoCrNi)OOH, conventional AEM pathway was monitored through the following pathway (Steps 6-9):

$$* + OH^{-} \rightarrow *OH + e^{-} \text{ (Step 6)}$$

$$*OH + OH^{-} \rightarrow *O + H_2O(l) + e^{-} \text{ (Step 7)}$$

$$*O + OH^{-} \rightarrow *OOH + e^{-} \text{ (Step 8)}$$

$$*OOH + OH^{-} \rightarrow * + O_2(g) + H_2O(l) + e^{-} \text{ (Step 9)}$$

As such, the Gibbs free energy changes for each step can be calculated by Supplementary Equations (12-15):

$$\Delta G_1 = \Delta G_{*OH} - eU + \Delta G_{pH} \tag{12}$$

$$\Delta G_2 = \Delta G_{*O} - \Delta G_{*OH} - eU + \Delta G_{pH} \tag{13}$$

$$\Delta G_3 = \Delta G_{*OOH} - \Delta G_{*O} - eU + \Delta G_{pH} \tag{14}$$

$$\Delta G_4 = 4.92 - \Delta G_{*OOH} - eU + \Delta G_{pH} \tag{15}$$

The Gibbs free energy differences of various intermediates were determined by Supplementary Equations (16-18):

$$\Delta E_{*OH} = E_{*OH} - E_{*} - (\mu_{H2O(l)} - 1/2\mu_{H2(g)}) \tag{16}$$

$$\Delta E_{*O} = E_{*O} - E_{*} - (\mu_{H2O(l)} - \mu_{H2(g)}) \tag{17}$$

$$\Delta E_{*OOH} = E_{*OOH} - E_{*} - (2\mu_{H2O(l)} - 3/2\mu_{H2(g)}) \tag{18}$$

To compare the energetically preferential pathway on different models, we also simulated the AEM pathway on (FeCoCrNi)OOH model and the LOM pathway on (CoCrNi)OOH model through the same computational method, respectively. For AEM pathway on (FeCoCrNi)OOH model, the pre-absorbed OH^{-} on Ni-Co dual-site (*i.e.*, Ni-μ-O_aH-Co motif) was directly deprotonated to from *O_a species, and then formed *O_aO_aH species (see Supplementary Fig. 39). For LOM pathway on (CoCrNi)OOH model, η-Ni-O_lH motif was deprotonated rather than adsorbed hydroxyl, and then proceeded nucleophilic attack of OH^{-} to form η-Ni-O_lO_aH motif, as schemed in Supplementary Fig. 40a.

Supplementary Note 6 | Consideration of other catalytic centers (Fe-neighboring Ni-Ni dual-site) on (FeCrNi)OOH model.

According to the simulated configurations, the first step for the pristine (FeCrNi)OOH model should be the deprotonation of Ni-η-O_lH, and form an Fe-(μ-O_lO_l)-Ni motif after structural relaxation (shown in Fig. 4a). This convergent configuration demonstrates that oxygen coupling spontaneously occurs at Fe-Ni site to form O_2 molecule, meaning Fe-Ni dual-site as catalytic center. To strengthen this argument, we have also considered other possible configurations with different catalytic center. For instance, after Ni-η-O_lH deprotonation, we compulsively constructed the O-O bond via two Ni-O motif (see Supplementary Fig. 36a, left configuration) to check whether Ni-Ni dual-site can also serve as catalytic center to stabilize O-O bond. Unfortunately, we find that this compulsively constructed O-O bond at Ni-Ni dual-site is highly unstable and cannot achieve the force and energy convergence. Moreover, the O-O bond undergoes spontaneous dissociation during the structural relaxation (Supplementary Fig. 36a, right configuration). This simulation result indicates that Ni-Ni dual-site cannot serve as catalytic center to furnish oxygen coupling.

Furthermore, to eliminate the influence of hydrogen atoms, we have also intentionally deleted all hydrogen atoms to expose M-O bond on the surface, carry out the structural relaxation to seek the energetically favorable configuration. As demonstrated in Supplementary Fig. 36b, oxygen coupling still occurs at Fe-Ni dual-site to form Fe-(μ-O_lO_l)-Ni motif after structural relaxation. Combining the computational results, we can conclude that Fe-Ni dual-site indeed facilitates oxygen coupling to form O-O bond. Thus, it is reasonable to regard it as ultimate catalytic center.

Supplementary Note 7 | Further statements of non-concerted electron-proton transfer step in LOM pathway.

Typically, OER process goes through the concerted electron-proton transfer step, in which the energy barrier of step is independent to proton concentration in the electrolyte as shown in Supplementary Fig. 42a. However, it has been widely reported that the non-concerted electron-proton transfer step will be involved when lattice oxygen ligands participate into oxygen evolution[29,30], namely, LOM pathway (Supplementary Fig. 42b). The origin of this unconventional step is the mismatch between the electron transfer kinetics and hydroxide affinity at the oxide/electrolyte interface. The downshifted *d*-band of Ni^{4+} species falls below the O_2/H_2O redox couple and improves the chemical potential of electrons, thereby lowering or even eliminating the barrier for electron transfer. As a result, the electron-proton transfer in RLS is decoupled, exhibiting a pH-correlated OER activity. As described in Supplementary Fig. 42b (a typical proposal of non-concerted electron-proton transfer cycling), the RLS is only a chemical step while O_2 desorption will contain the electron transfer (*i.e.*, $OO^* \rightarrow * + O_2(g) + e^-$).

Supplementary References

1 Lee, S., Banjac, K., Lingenfelder, M. & Hu, X. Oxygen isotope labeling experiments reveal different reaction sites for the oxygen evolution reaction on nickel and nickel iron oxides. *Angew. Chem. Int. Ed.* **58**, 10295-10299 (2019).

2 Wagner, C. D., Riggs, W. M., Davis, L. E. & Moulder, J. F. Handbook of X-ray Photoelectron spectrscopy. (Published by Perkin-Elmer Corporation, Physcial Electronics Division, U. S. A., 1979).

3 Bard, A. J., Parsons, R. & Jordan, J. Standard potentials in aqueous solution. (CRC Press, Boca Raton, 1985).

4 McCrory, C. C. L., Jung, S., Peters, J. C. & Jaramillo, T. F. Benchmarking heterogeneous electrocatalysts for the oxygen evolution reaction. *J. Am. Chem. Soc.* **135**, 16977-16987 (2013).

5 Song, F. & Hu, X. Exfoliation of layered double hydroxides for enhanced oxygen evolution catalysis. *Nat. Commun.* **5**, 4477 (2014).

6 Fan, K. *et al.* Nickel-vanadium monolayer double hydroxide for efficient electrochemical water oxidation. *Nat. Commun.* **7**, 11981 (2016).

7 Zhao, S. *et al.* Ultrathin metal-organic framework nanosheets for electrocatalytic oxygen evolution. *Nat. Energy* **1**, 16184 (2016).

8 Zhang, B. *et al.* Homogeneously dispersed multimetal oxygen-evolving catalysts. *Science* **352**, 333-337 (2016).

9 Fan, L. *et al.* 3D Core-shell NiFeCr catalyst on a Cu nanoarray for water oxidation: synergy between structural and electronic modulation. *ACS Energy Lett.* **3**, 2865-2874 (2018).

10 Yan, Z. *et al.* Anion insertion enhanced electrodeposition of robust metal hydroxide/oxide electrodes for oxygen evolution. *Nat. Commun.* **9**, 2373 (2018).

11 Jiang, J. *et al.* Atomic-level insight into super-efficient electrocatalytic oxygen evolution on iron and vanadium co-doped nickel (oxy)hydroxide. *Nat. Commun.* **9**, 2885 (2018).

12 Cheng, W. *et al.* Lattice-strained metal-organic-framework arrays for bifunctional oxygen electrocatalysis. *Nat. Energy* **4**, 115-122 (2019).

13 Han, X.-B. *et al.* Ultrasmall abundant metal-based clusters as oxygen-evolving catalysts. *J. Am. Chem. Soc.* **141**, 232-239 (2019).

14 Cai, Z. *et al.* Amorphous nanocages of Cu-Ni-Fe hydr(oxy)oxide prepared by photocorrosion for highly eficient oxygen evolution. *Angew. Chem. Int. Ed.* **58**, 4189-4194 (2019).

15 Gao, Z.-W. *et al.* Engineering NiO/NiFe LDH intersection to bypass scaling relationship for oxygen evolution reaction via dynamic tridimensional adsorption of intermediates. *Adv. Mater.* **31**, 1804769 (2019).

16 Xue, Z. *et al.* Interfacial electronic structure modulation of NiTe nanoarrays with NiS nanodots facilitates electrocatalytic oxygen evolution. *Adv. Mater.* **31**, 1900430 (2019).

17 Chen, J. Y. C. *et al.* Operando analysis of NiFe and Fe oxyhydroxide electrocatalysts for water oxidation: detection of Fe^{4+} by Mössbauer spectroscopy. *J. Am. Chem. Soc.* **137**, 15090-15093 (2015).

18 Suntivich, J. *et al.* Design principles for oxygen-reduction activity on perovskite oxide catalysts for fuel cells and metal–air batteries. *Nat. Chem.* **3**, 546-550 (2011).

19 Forslund, R. P. *et al.* Exceptional electrocatalytic oxygen evolution via tunable charge transfer interactions in $La_{0.5}Sr_{1.5}Ni_{1-x}Fe_xO_{4\pm\delta}$ Ruddlesden-Popper oxides. *Nat. Commun.* **9**, 3150 (2018).

20 Lee, J. G. *et al.* A new family of perovskite catalysts for oxygen-evolution reaction in alkaline media: $BaNiO_3$ and $BaNi_{0.83}O_{2.5}$. *J. Am. Chem. Soc.* **138**, 3541-3547 (2016).

21 Li, X. *et al.* Exceptional oxygen evolution reactivities on CaCoO3 and SrCoO3. *Sci. Adv.* **5**, eaav6262 (2019).

22 Yagi, S. *et al.* Covalency-reinforced oxygen evolution reaction catalyst. *Nat. Commun.* **6**, 8249 (2015).

23 Shin, H., Xiao, H. & Goddard, W. A. In silico discovery of new dopants for Fe-doped Ni oxyhydroxide (Ni1–xFexOOH) catalysts for oxygen evolution reaction. *J. Am. Chem. Soc.* **140**, 6745-6748 (2018).

24 Bajdich, M., García-Mota, M., Vojvodic, A., Nørskov, J. K. & Bell, A. T. Theoretical investigation of the activity of cobalt oxides for the electrochemical oxidation of water. *J. Am. Chem. Soc.* **135**, 13521-13530 (2013).

25 Martirez, J. M. P. & Carter, E. A. Unraveling oxygen evolution on iron-doped β-nickel oxyhydroxide: the key role of highly active molecular-like sites. *J. Am. Chem. Soc.* **141**, 693-705 (2019).

26 Nørskov, J. K. et al. Origin of the overpotential for oxygen reduction at a fuel-cell cathode. *J Phys. Chem. B* **108**, 17886-17892 (2004).

27 Rossmeisl, J., Logadottir, A. & Nørskov, J. K. Electrolysis of water on (oxidized) metal surfaces. *Chem. Phys.* **319**, 178-184 (2005).

28 Peterson, A. A., Abild-Pedersen, F., Studt, F., Rossmeisl, J. & Nørskov, J. K. How copper catalyzes the electroreduction of carbon dioxide into hydrocarbon fuels. *Energy Environ. Sci.* **3**, 1311-1315 (2010).

29 Huang, Z.-F. *et al.* Chemical and structural origin of lattice oxygen oxidation in Co-Zn oxyhydroxide oxygen evolution electrocatalysts. *Nat. Energy* **4**, 329-338 (2019).

30 Grimaud, A. *et al.* Activating lattice oxygen redox reactions in metal oxides to catalyse oxygen evolution. *Nat. Chem.* **9**, 457-465 (2017).
